# Refocusing algorithmic fairness on feature-level bias: A diagnostic approach using dutch EHR data

**DOI:** 10.1371/journal.pdig.0001105

**Published:** 2026-10-26

**Authors:** Cathleen S. Parsons, Shelley-Ann M. Girwar, Sepinoud Azimi, Marco R. Spruit, Marcel R. Haas

**Affiliations:** 1 Department of Engineering Systems & Services, Faculty of Technology, Policy and Management, Delft University of Technology, Delft, The Netherlands; 2 Department of Public Health and Primary Care, Leiden University Medical Centre, The Hague, The Netherlands; 3 Leiden Institute of Advanced Computer Science (LIACS), Faculty of Science, Leiden University, Leiden, The Netherlands; University of Exeter, UNITED KINGDOM OF GREAT BRITAIN AND NORTHERN IRELAND

## Abstract

While algorithmic fairness research in healthcare has predominantly focused on disparities in model performance, less attention has been given to the underlying data structures that may drive such disparities. High-level fairness metrics often obscure the deeper feature-level dynamics necessary for a critical and context-aware assessment of fairness. To address this gap, we propose and apply a diagnostic framework termed Feature-Level Bias Identification And Sensemaking (FL-BIAS). As a case study, we conducted a secondary analysis of a retrospective cross-sectional cohort study using electronic health record data from Dutch general practitioners, linked with sociodemographic data from Statistics Netherlands. The dataset included 112,872 patients, of whom 16.2% had a non-Western migration background. Hospitalization in the following year was modeled using Johns Hopkins Aggregated Diagnosis Groups (ADGs). We trained logistic regression and XGBoost models on different subgroup datasets to evaluate performance disparities using fairness metrics. To analyze feature-level contributions to predictions and errors, we applied Kernel Shapley Values and Cohort Shapley methods. Exploratory analysis revealed significant differences in socioeconomic status (SES) and ADG distributions between Dutch and non-Western groups, though Multiple Correspondence Analysis showed minimal structural variation. Mediation analysis indicated that the effect of migration background on hospitalization was largely mediated by SES, with potential unobserved confounding. While standard fairness metrics indicated modest bias in favor of non-Western patients, deeper feature-level analyses revealed subgroup-specific patterns of variable importance that suggest potentially less favorable underlying conditions. For instance, malignancy (ADG 32) had a stronger predictive impact among non-Western patients but contributed less to false negatives compared to Dutch patients, possibly reflecting structural disparities in cancer diagnosis and care. These findings highlight the need for contextual, multi-level evaluations of algorithmic bias. Fairness in healthcare algorithms must be approached as a socio-technical challenge, requiring multidisciplinary collaboration to uncover root causes in the processes that generate the data.

## Author summary

Unfair algorithms often originate in the data itself. To help detect such hidden biases, we combined known data science methods into a diagnostic approach named Feature-Level Bias Identification And Sensemaking (FL-BIAS). Using Dutch general practitioner records linked with national demographic information, we explored how patient migration background, after controlling for socioeconomic status affects predictions of hospital admissions. We discovered that certain medical conditions, such as cancer and chronic illness, contributed differently to predictions for Dutch compared to non-Western patients, revealing subtle but important patterns in how data reflects social inequalities. Our goal was to better understand how inequalities can be hidden in health data, supporting the design of fairer and more transparent healthcare AI tools that truly serve all patients.

## Introduction

The field of algorithmic fairness remains conceptually unsettled. Despite ongoing debates over how fairness should be defined, which metrics should be prioritized, and which mitigation strategies are most effective [1–3], there is widespread recognition that deploying inadequately evaluated algorithmic models introduces serious risks, particularly in high-stakes domains such as healthcare [4,5]. Predictive models that perform differently across subpopulations risk reinforcing or exacerbating existing societal disparities, a phenomenon termed algorithmic bias [6]. In response, numerous guidelines and ethical frameworks have emerged that position fairness as a central design principle, including the World Health Organization’s *Ethics and Governance of Artificial Intelligence for Health* [7], the European Union’s AI Act [8], and other national-level initiatives.

Over the past decade, substantial research on algorithmic fairness in healthcare has primarily focused on the definitional complexities of fairness and on developing technical bias mitigation strategies such as reweighting, resampling, fairness-aware training, threshold adjustment and calibration techniques [2,3,9]. However, as Corbett-Davies et al. have argued [10], formal fairness definitions often fail to address structural disparities when applied without a nuanced, contextual understanding, potentially leading to decision policies that result in adverse outcomes. Therefore, fairness approaches need to be explicitly outcome-oriented and context-aware [10]. This is particularly crucial in health applications, where sensitive attributes may serve as proxies for socioeconomic status (SES) [11,12]. For example, one study found that Black race was not associated with higher in-hospital mortality compared to White race, after adjusting for socio-demographic and patient health profiles upon admission [13]. Yet such context dependent relationships are frequently omitted from fairness analyses, where mitigation strategies often result in suboptimal solutions [3,14].

Another complication concerns the effectiveness of bias mitigation strategies, which depends heavily on the specific dataset and model context [11,15,16]. A well-grounded understanding of relationships between features within the dataset can even help to overcome the trade-off between fairness and accuracy when applying mitigation strategies that modify the data. Leininger et al. demonstrate that causal pre-processing methods, which aim to approximate a counterfactual “fair world” where sensitive features are causally independent of the outcome, can improve fairness without negatively impacting accuracy by addressing bias at its structural source [17].

Both the dependency of fairness definitions on data context and the reliance of mitigation strategies on dataset structure highlight the need to understand where and how bias manifests in the data. Fairness methodologies are thus inherently vulnerable to the validity of assumptions made by human experts, including beliefs about relationships between features and outcomes, as well as the broader context in which the data was generated, such as the presence of proxy variables. To support domain experts in identifying patterns that require interpretation, and to inform the development of appropriate bias mitigation strategies and interventions, a systematic examination of feature behavior across subpopulations is essential, yet this step seems often overlooked.

Prior fairness assessments of electronic health record (EHR) data have largely focused on applying group-level fairness metrics rather than uncovering potential sources of bias at the feature level [2,18]. Alongside fairness metrics, complementary methods such as Shapley values, commonly used in explainable AI, can help reveal differences in feature responsiveness to prediction risks across subgroups in fairness analyses [19–21]. Therefore, the objective of this study is to propose and apply a diagnostic approach to assess feature-level bias in predictive models trained on EHR data. A systematic examination of feature behavior offers a path beyond fairness metrics toward a human-in-the-loop assessment of the extent and nature of algorithmic bias.

As a case study, we compared model performance and variable behavior between individuals from Dutch and non-Western migration backgrounds, controlling for SES, in Dutch primary care EHR data. A recent survey revealed that some Dutch patients with non-Western migration backgrounds perceive their health concerns as inadequately addressed by healthcare providers, potentially due to ethnic bias [22]. This finding motivated the examination of whether such disparities are reflected in Dutch EHR data when developing predictive models. Through this case study, we successfully identified subgroup-specific differences in feature behavior that were not captured by standard fairness metrics.

## Methods

### Diagnostic approach

Fig 1 illustrates the proposed diagnostic approach Feature-Level Bias Identification and Sensemaking (FL-BIAS), which systematically assesses underlying data structures and feature behavior between subgroups. Interpreting data differences as indicators of algorithmic bias requires, as in causal inference, both data patterns and contextual understanding. Starting with a general understanding of the dataset’s structure, successive steps progressively uncover the features behind algorithmic bias.

**Fig 1:**
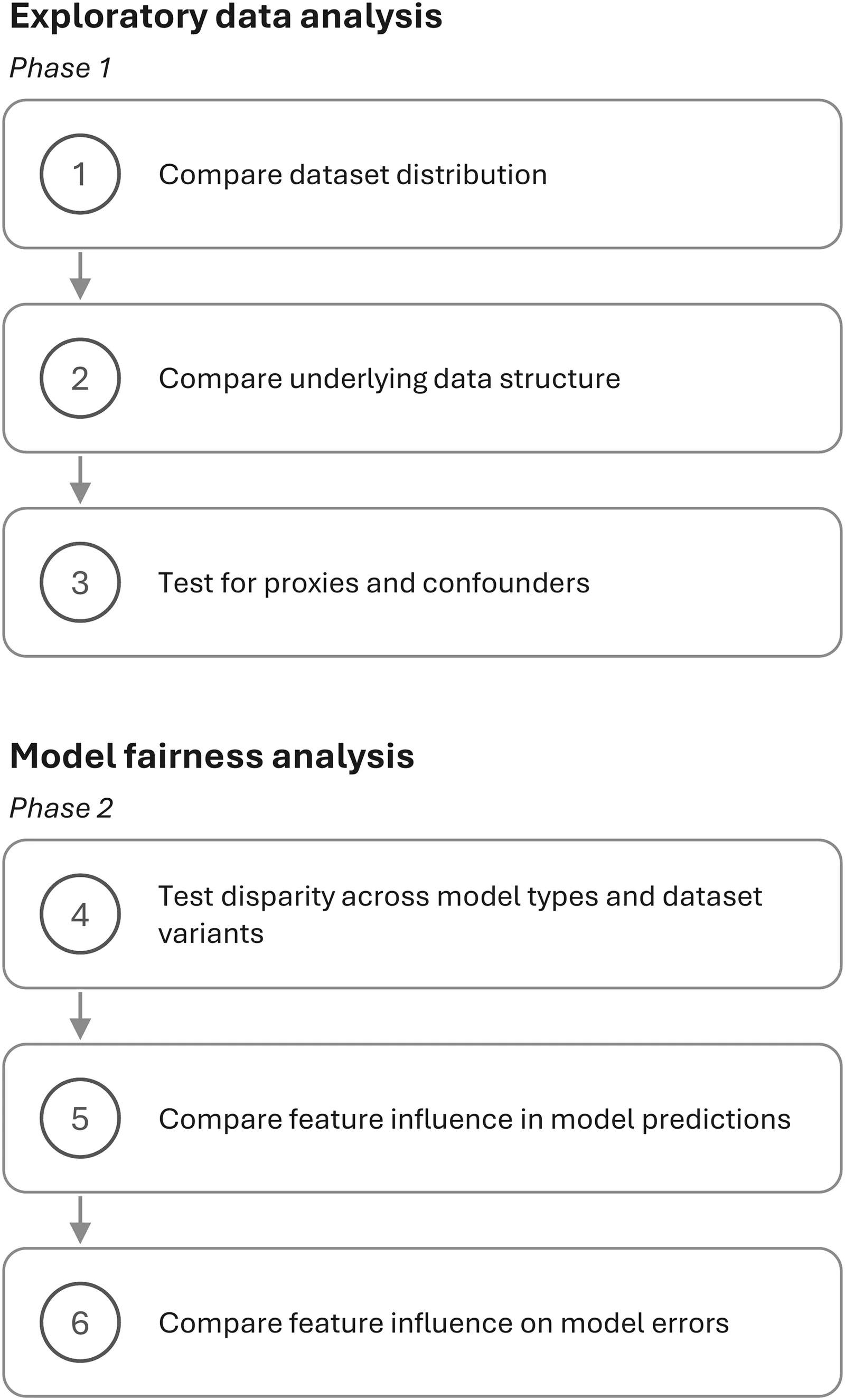
FL-BIAS (Feature-Level Bias Identification And Sensemaking): A diagnostic framework that systematically assesses underlying data structures and feature behavior between subgroups.

### Exploratory data analysis phase

The first phase provides a systematic examination of the datasets for the two subgroups.

Step 1: Evaluate subgroup distributions of demographic and clinical features to identify imbalances that may explain observed differences in model performance. This can include feature prevalence analysis, statistical tests, and visual inspections.

Step 2: Explore how features contribute to the overall data structure and how they co-occur, revealing relationships that could influence predictions. This may involve Multiple Correspondence Analysis (MCA) or Principal Component Analysis (PCA).

Step 3: Account for potential confounders to correctly interpret the effect of sensitive attributes on outcomes and to determine which variables should be included in prediction models. Possible approaches include Causal Mediation Analysis or constructing data-driven Directed Acyclic Graphs (DAGs).

### Model fairness analysis phase

The second phase focuses on model evaluation and identifying the extent of model performance bias at the feature level.

Step 4: Develop and evaluate models across multiple subdatasets and use diverse performance metrics to detect subgroup-specific disparities. Compare metrics such as Equalized Odds, False Negative/Positive rates, calibration curves, and ROC-AUC across groups to identify performance gaps.

Step 5: Assess relative feature importance differences between subgroups to identify uneven model dependencies. For instance, by comparing coefficients or permutation-based feature importances, or by applying XAI techniques such as Kernel Shapley Values to quantify how features contribute differently to predictions across groups.

Step 6: Examine model prediction errors across groups to identify which features drive observed disparities. This can involve analyzing misclassification patterns by feature values or using error attribution analysis such as Cohort Shapley Values.

The total picture from these steps can reveal inequalities in the datasets that may reflect inequities between subgroups after consideration of omitted variables, unobserved confounding, and the social and medical processes that generate the data. Whether a given inequality reflects an inequity is a question of interpretation, requiring clinical and social context. The following case study describes how this diagnostic framework was applied to real-world data, outlining the methods used to operationalize each above mentioned step.

### Case study design

This study was a secondary analysis of a retrospective cohort study [23,24]. The EHR data from Dutch general practices, covering the period from January 2016 to December 2016, were linked in a controlled environment with data from Statistics Netherlands, the Dutch central bureau for statistics (CBS). These included 2016 data on migration background and SES, as well as 2017 data on hospitalization outcomes.

### Study population

General practices across four diverse and representative regions in the Netherlands provided data from their patient EHRs containing ICPC-1 diagnosis codes (Dutch version of International Classification of Primary Care) and medication prescriptions (Anatomical Therapeutic Chemical codes, ATC-4). To reduce data dimensionality, the Johns Hopkins Adjusted Clinical Groups (ACG) system, version 12.1, was used to categorize ICPC-1 diagnosis codes into 32 Aggregated Diagnosis Groups (ADGs) based on comparable levels of burden of care by capturing complexity and intensity of health conditions [25]. As prescribing behavior, typically guided by well-established treatment protocols, may diverge from diagnostic entries in electronic health records, the ACG system was additionally used to generate 81 Rx-defined Morbidity Groups (Rx-MGs) from prescription data. This dual approach has been used in previous studies [26,27], and is applied here to identify distinct sources of potential bias in EHR data by comparing models developed from diagnostic data with those based on prescription data.

Our EHR data were linked with three datasets from Statistics Netherlands: 2017 hospitalization data and 2016 data on migration background and SES. For migration background, a dataset was used that assigns each subject in the Netherlands to either their own country of birth or, if born in the Netherlands, to the country of birth of their non-Dutch parent (with the mother’s country used by default if both parents are non-Dutch). Although this classification has been subject to public debate [28,29], it reflects the categorization used by Statistics Netherlands in 2016, on which the linked data are based. This categorization has also been used in a previous Dutch study researching the relation between ethnicity and population cardiovascular health [30]. For our own analysis, we divided the database, which contained no missing values, into two groups: Dutch and non-Western country codes with at least 500 people per country, ten countries in total. Patients with a Western migration background were excluded to maintain a clear analytical contrast between the study groups that formed the focus of the fairness analysis.

For SES, similar to the approach used in a previous Dutch population-based study [30], we used data based on the percentile ranking of household prosperity, which included standardized disposable household income and wealth. CBS data on occupation and education were not included in the analysis of SES, as they contained a high degree of missing data (30%). Following that previously published approach [30], we categorized the lowest 20% as low SES, the highest 20% as high SES, and the middle 60% as middle SES, using the distribution in the total dataset that includes participants with Western backgrounds. The SES variable had only 0.6% missing data, which were imputed using the mode.

Due to the imbalanced nature of subgroups of the EHR dataset, a subset with an equal distribution of Dutch and non-Western populations was created as an additional dataset for comparative analyses. Furthermore, given that many patients are relatively healthy and thus have low rates of hospitalisation, a subset of high-morbidity patients (i.e., those with at least one major ADG diagnosis) was constructed for subgroup-level analysis.

### Analysis

As outlined in Fig 1, we first compared the prevalence distributions of ADGs between Dutch and non-Western populations. Second, to assess the underlying data structure, we performed a Multiple Correspondence Analysis (MCA) using the ‘FactoMineR’ and ‘factoextra’ R packages. Both sub-datasets had an equal distribution of the confounders age, sex, SES and hospitalization outcomes. MCA reduces categorical data to latent dimensions that capture variance in feature associations, and visualizes how categories cluster, thereby revealing potential co-occurrence patterns. Squared cosine (cos^2^) values are added, which measures the degree of association and indicate how well each feature is represented by the first two dimensions. Third, we conducted a mediation analysis with the ‘Mediation’ R package [31] to examine the extent to which the effect of migration background on hospitalization was mediated by SES. Sex and age are potential confounders influencing SES and hospitalization risk and therefore included as co-variates. Clinical risk factors, in our context ADGs, were not included, as they are considered to lie on the causal pathway from migration background to hospitalization, potentially operating through SES, and including them would block part of the mediating effect. Causal mediation analysis relies on the assumption of sequential ignorability [31], which requires the absence of unmeasured confounders and necessitates sensitivity analysis. We choose as the sensitivity parameter (ρ) for unobserved confounding the correlation between the residuals of the mediator and outcome regressions [31]. The sensitivity analysis was conducted by varying the value of ρ in increments of 0.1.

For the Model Fairness Analysis Phase of our framework (Fig 1), we developed multiple models as outlined in Fig 2. To control for potential confounding effects, age, sex and SES were incorporated as variables in our models. We selected hospitalization (prevalence of 10% in our dataset) in the following year, one of the standard predictions by the ACG system, as the outcome variable. We selected logistic regression as our baseline model for predicting hospitalization. Logistic regression is a commonly used modelling approach in ACG-based research [23,32–37], including studies conducted by the Johns Hopkins ACG developers [38,39]. To account for any non-linear relationships and interactions between the features, we compared the logistic regression model to a XGBoost model, since gradient-boosted trees are among the best-performing methods for tabular prediction tasks [40]. Additionally, a logistic regression model utilizing only eight major ADGs [3,4,9,11,16,22,25,32], each associated with high demand for healthcare services [41], was developed. Major ADG models are commonly employed in practice and offer the advantage of reducing data dimensionality and improving explainability. Model specifications are detailed in S1 Table. During development, no threshold adjustments were applied, as the primary aim was not to optimize predictive performance for imbalanced datasets but to examine differences in model behavior between the two subgroups using identical model architecture.

**Fig 2:**
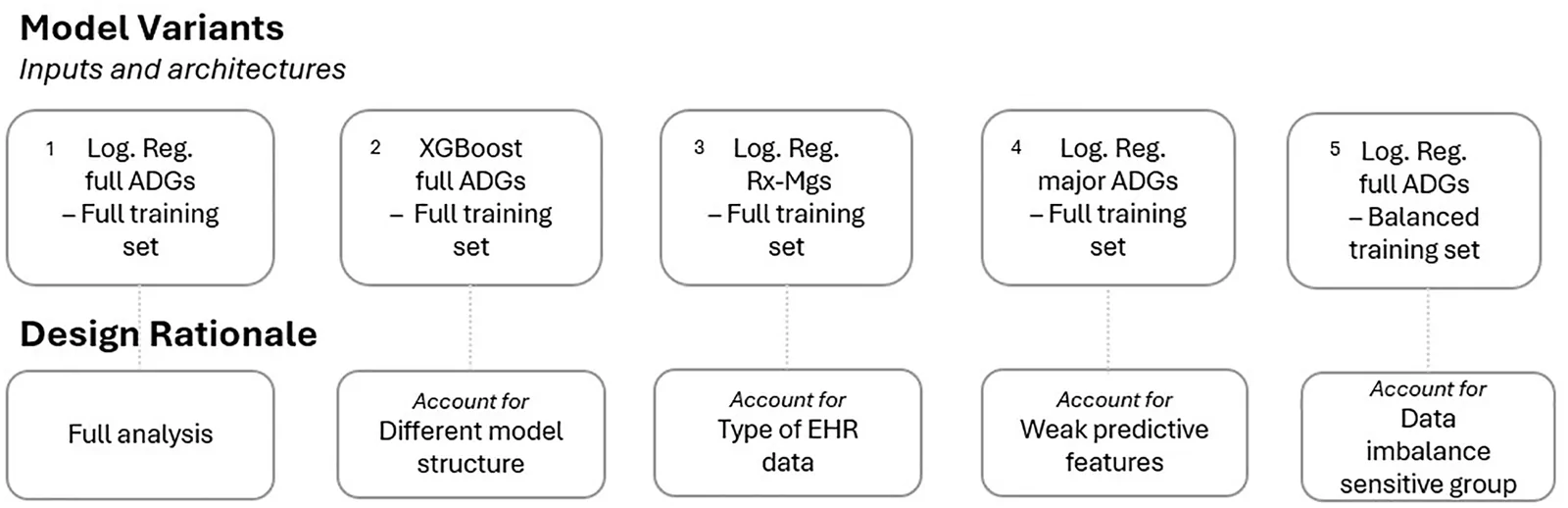
Overview model variants (Inputs + Architectures) and its rationale.

The models in Fig 2 were assessed on several performance metrics detailed in the following section. In addition, the LR–32 ADGs, LR–balanced dataset, and RX-MG models were evaluated on Dutch-only and non-Western-only datasets. Feature importance differences were examined specifically for the LR–32 ADGs model, both on the full dataset and in subgroup-specific models for Dutch and non-Western patients. To ensure reliable coefficient interpretation in these logistic regression models, multicollinearity analyses were performed using the Variance Inflation Factor (VIF) and correlation heatmaps. Lastly, we conducted analyses using Shapley Values (SV). SV are being increasingly used in the algorithmic fairness field to quantify contributions of individual variables to algorithmic bias [19,42]. For this analysis, only major ADGs were selected to limit computational expense. We calculated two types of Shapley Values: Kernel Shapley Values, implemented using the ‘Shapr’ package in R [43], and Cohort Shapley Values, implemented using the ‘cohort shapley’ Python package [42]. The *shapr* implementation was used to compare feature contributions to model predictions between the Dutch and non-Western datasets. It computes dependence-aware Shapley values by estimating conditional expectations instead of assuming feature independence. Feature dependencies were accounted for using the *ctree* approach [44], which applies conditional inference trees to approximate conditional feature distributions. Cohort Shapley was used to measure SV to instances where the model predicted no event but one actually occurred (false negatives) and for the differences between observed and predicted outcomes (residuals). This method by [21,42] uses only observed combinations in data, thereby avoiding unrealistic feature combinations, and accounts for both feature interactions and dependencies.

### Outcome

Fairness can be defined in multiple ways that are theoretically incompatible when outcome prevalence differs across groups [45]. Overall, a distinction is drawn between whether the desired outcome involves equal performance or distributive justice, which refers to equal allocation. In the setting of this study, it is preferred that the model performs comparably across all groups, which is generally used for predictive models in the healthcare field. For predicting hospitalisation, we are particularly interested in false negatives rates (FNR) as metric, since it can be used to measure undertreatment in the minority groups [46]. Equalized odds (EO) [47] is a commonly used fairness metric, ensuring that true positive rates and false positive rates are balanced among subgroups. An EO ratio of 1 indicates complete fairness. EO was calculated via the Fairlearn package [48]. Fairness metrics were computed on the complete test set using the Fairlearn toolkit, following their recommended approach for subgroup analysis.

To evaluate 5-fold cross-validation model performance on an imbalanced dataset, we included several additional metrics in addition to FNR, accuracy and the C-statistic. These included Precision (TP / [TP + FP]) and the F1-score (2 × Precision × Recall / [Precision + Recall]), which balances the trade-off between Precision and Recall (TP / [TP + FN]). The Matthews Correlation Coefficient (MCC) was also reported, as it is particularly well-suited for imbalanced data. MCC considers all four quadrants of the confusion matrix, True Positives (TP), True Negatives (TN), False Positives (FP), and False Negatives (FN), proportionally considering both the positive and negative elements within the dataset. It is defined as: ((TP × TN-FP × FN)/ √((TP + FP)×(TP + FN)×(TN + FP)×(TN + FN))). The MCC ranges from –1 to +1, where +1 indicates perfect prediction, 0 indicates performance equivalent to random chance, and –1 indicates complete disagreement between predictions and observations.

### Ethical considerations

This analysis used the general practice data from a previous project [23,24]. For that project, the Medical Ethics Committee of Leiden University Medical Center (reference G19.048) determined that formal ethical review was not required, as it involved retrospectively and routinely collected, de-identified health data.

Processing special-category data such as health and migration background was carried out in accordance with GDPR Article 9(2)(j), which permits such processing for scientific research in the public interest when appropriate safeguards are in place. All analyses were performed exclusively within the Statistics Netherlands secured environment, and researchers had no access to directly identifiable data at any time. Patients who could not be linked to the Statistics Netherlands database and deceased patients, who could not exercise the right to object to data use, were excluded. The output of all analyses was subject to Statistics Netherlands disclosure control to ensure that all published results were sufficiently anonymized and posed no risk of re-identification.

## Results

### Step 1: Compare dataset distribution

The total study population of our case study consisted of 112,872 patients of which 16% were identified as non-Western and 84% as Dutch Table 1. A significant difference was observed in the SES of non-Western individuals, marked by a 20% prevalence percentage point difference to Dutch individuals. In terms of morbidity, the Dutch population, which was on average older, exhibited a higher prevalence of ADGs and a greater frequency of hospitalization.

**Table 1:** Patient characteristics in the retrospective dataset.

	Overall	Dutch	Non-Western
n (%)	112872 (100)	94628 (83.8)	18244 (16.2)
Age (mean (SD))	45.92 (19.58)	46.54 (19.78)	42.74 (18.20)
Male (%)	55356 (49.0)	46694 (49.3)	8662 (47.5)
SES (%)			
*High*	27627 (24.5)	25400 (26.8)	2227 (12.2)
*Middle*	71793 (63.6)	61093 (64.6)	10700 (58.6)
*Low*	12787 (11.3)	7596 (8.0)	5191 (28.5)
*NA*	665 (0.6)	539 (0.6)	126 (0.7)
GP visits (mean (SD))	3.43 (4.26)	3.40 (4.27)	3.62 (4.21)
Hospitalized (%)	10955 (9.7)	9290 (9.8)	1665 (9.1)

S2 Table provides an overview on the prevalence distribution of ADGs per population dataset. Comparing the prevalence in both datasets, ADG1, ADG 5, and ADG 7 had the largest difference, with more than 5% percentage prevalence point difference and being statistically significant.

### Step 2: Compare underlying data structure

Fig 3A and Fig 3B visually represent the contribution values for each ADG's presence and absence across the first two dimensions and their clustering. The plots represent a modest 15% of the dataset variance, consistent with high-dimensional categorical clinical data where variance is distributed across many components. The primary dimensions for both the Dutch and the non-Western dataset presented comparable but relatively low eigenvalues, indicating that no single combination of ADGs accounts for a large portion of the variability within the data (S1 File). Features with low cos² values, such as ADG + 17 and ADG + 18, are poorly represented by the first two dimensions. Therefore, any apparent shift in their positions between the Dutch and non-Western plots of Fig 3 should not be interpreted as meaningful. However, ADG + 14 shows a notable positional shift with a modest cos² value, suggesting different co-occurrence patterns between groups.

**Fig 3:**
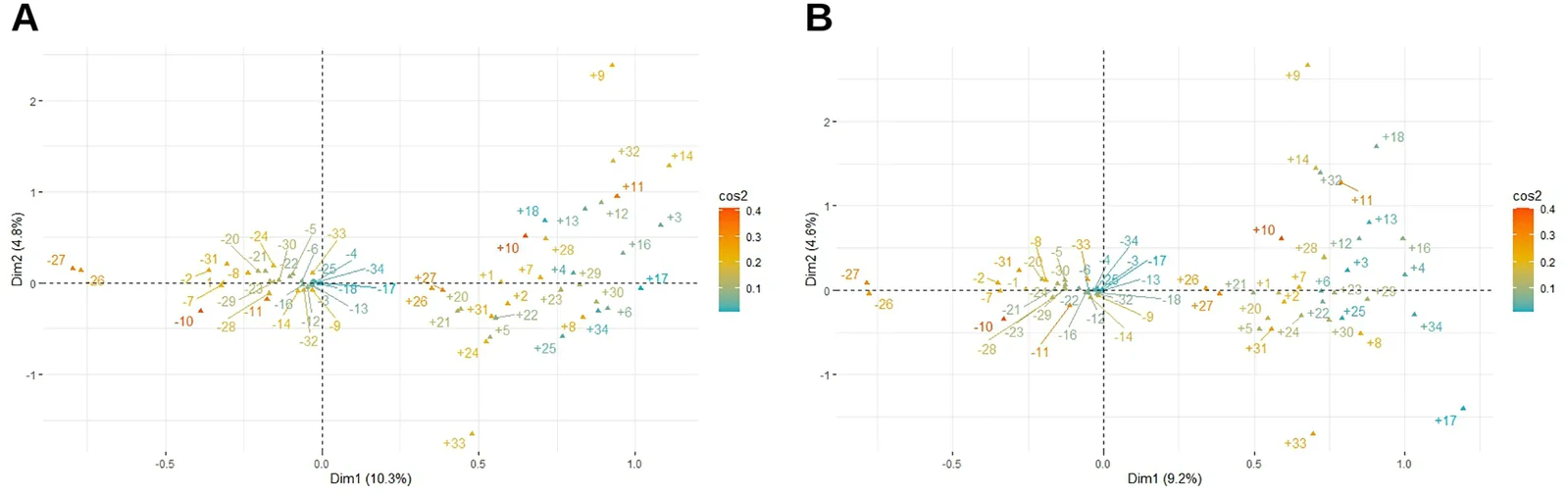
Multiple Correspondence Analysis of ADG variables in Dutch dataset (A) and non-Western dataset (B). Triangles indicate feature positions (co-occurrences), with “+” for ADG present and “-” for ADG absent. Cos^2^ values measures the degree of association and indicate how well each feature is represented by the first two dimensions.

### Step 3: Test for proxies & confounders

The Average Causal Mediated Effect (ACME), estimating whether non-Western background is associated with higher hospitalization rates through lower SES, was statistically significant for both the treated non-Western group (0.005695) and the control Dutch group (0.005758), with p-values less than 0.001. In contrast, the Average Direct Effect (ADE), which captures the direct effect of migration background on hospitalization after accounting for SES, was not statistically significant (p = 0.68). The proportion mediated exceeded 1.0 (≈1.35), consistent with full mediation and suggestive of a suppression effect, although this pattern is based on a non-significant direct effect. In sensitivity analysis, the mediation effect (ACME) was eliminated when ρ reached −0.1, revealing that a small degree of unobserved confounding could nullify the mediation effect. Additional estimation details are provided in S3 Table.

Overall, the mediation analysis indicates that migration background does not directly affect hospitalization. Instead, the statistically significant but small disparities appear to be fully explained by socioeconomic differences, with possible contributions from other unmeasured factors.

### Step 4: Test for disparity across model types and dataset variants

An overview of the performance metrics for the models can be found in Table 2. Despite correction for imbalanced data during model training, the performance of each model, particularly the XGBoost, was found to be suboptimal. This is primarily demonstrated by the low precision scores. Given that 90% of the patients were not hospitalized and major ADGs are associated with higher risk of hospitalization, further analysis was conducted on a sub-dataset consisting exclusively of patients with high morbidity, identified by the presence of one or more major ADGs. While this approach enhanced precision, it also led to a slightly higher algorithmic bias for both the full model and the major ADG model. This increase in disparity also occurred when reducing the model ADG variables to the eight major ADGs.

For the models based on ADG variables, the EO ratio was below one and non-Western individuals had a slightly higher FNR. This disparity effect remained when the models were trained on the balanced dataset. Overall, the logistic regression ADG-32 model demonstrated slightly better performance metrics for the non-Western population (Table 2), as reflected in a Matthews parity value of 1.051 (S4 Table) and illustrated by ROC curve analysis for each subgroup (Fig 4). By contrast, the logistic model trained on medication prescription data (Rx-MG) exhibited a lower degree of disparities and demonstrated a slightly more favorable performance when applied to the Dutch population (Table 2, Fig 4).

**Fig 4:**
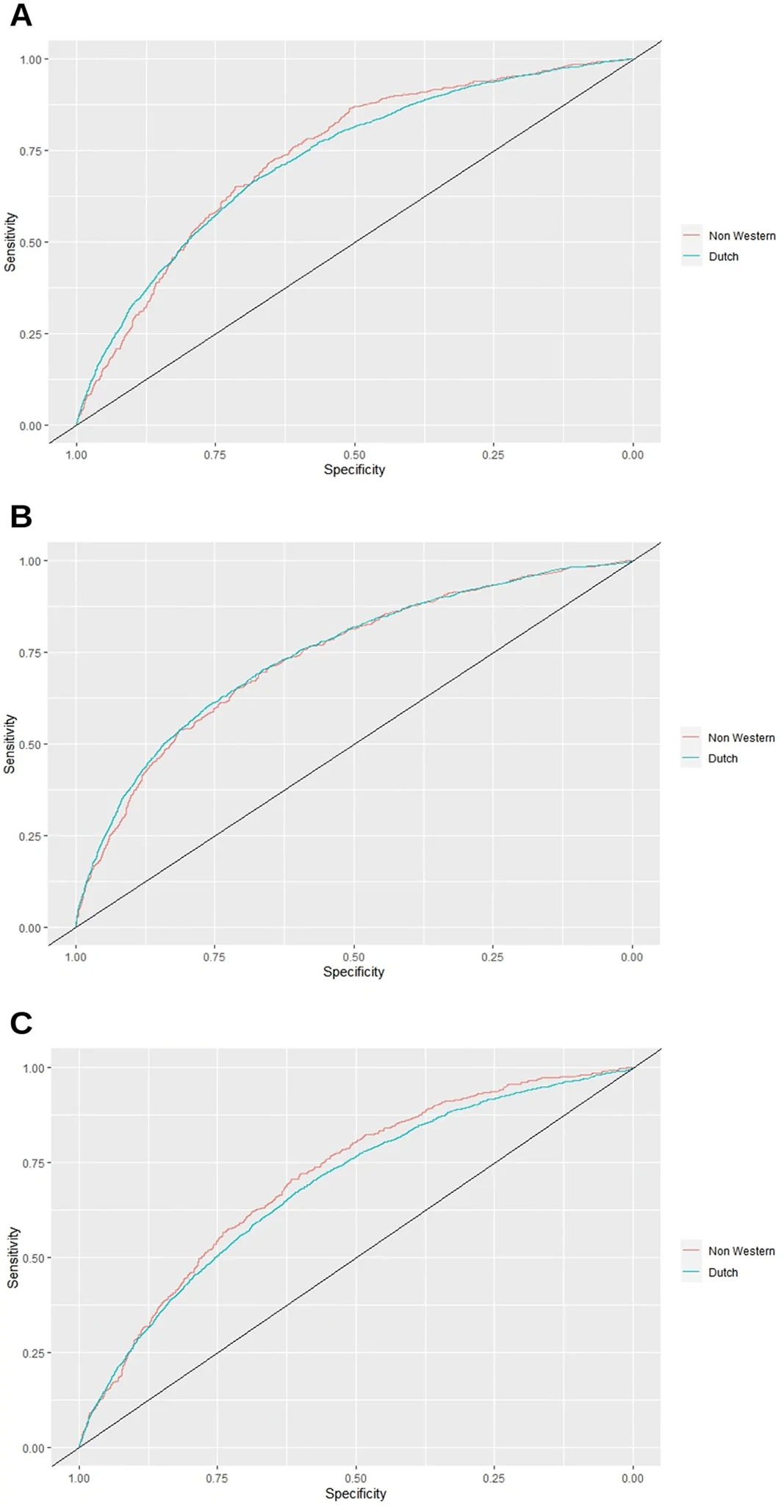
ROC curves of the logistic regression model with 32 ADG (A), the logistic regression model with Rx-MG (B), and the XGBoost model with 32 ADG (C).

### Step 5: Compare feature influence and responsiveness in model predictions

The VIF analysis showed minimal multicollinearity among ADG features, with a mean VIF of 1.094 (SD = 0.075) (S5 Table). This was supported by the low pairwise correlations between ADGs (S1 Fig), where the highest observed correlations were between ADG 10 & 11, ADG 26 & 27 and ADG 7 & ADG 11. Logistic regression coefficients (S5 Table) identified ADG 33 (Pregnancy), ADG 32 (Malignancy), ADG 11 (Chronic Medical: Unstable), and ADG 9 (Likely to Recur: Progressive) as the strongest predictors of hospitalization. With the exception of pregnancy, these ADGs belong to the Major ADG category. Kernel Shapley Value analysis (Fig 5; S6 Table) of the major ADG model further highlighted the importance of ADG 11, ADG 32, and ADG 9 in the model’s predictions.

**Fig 5:**
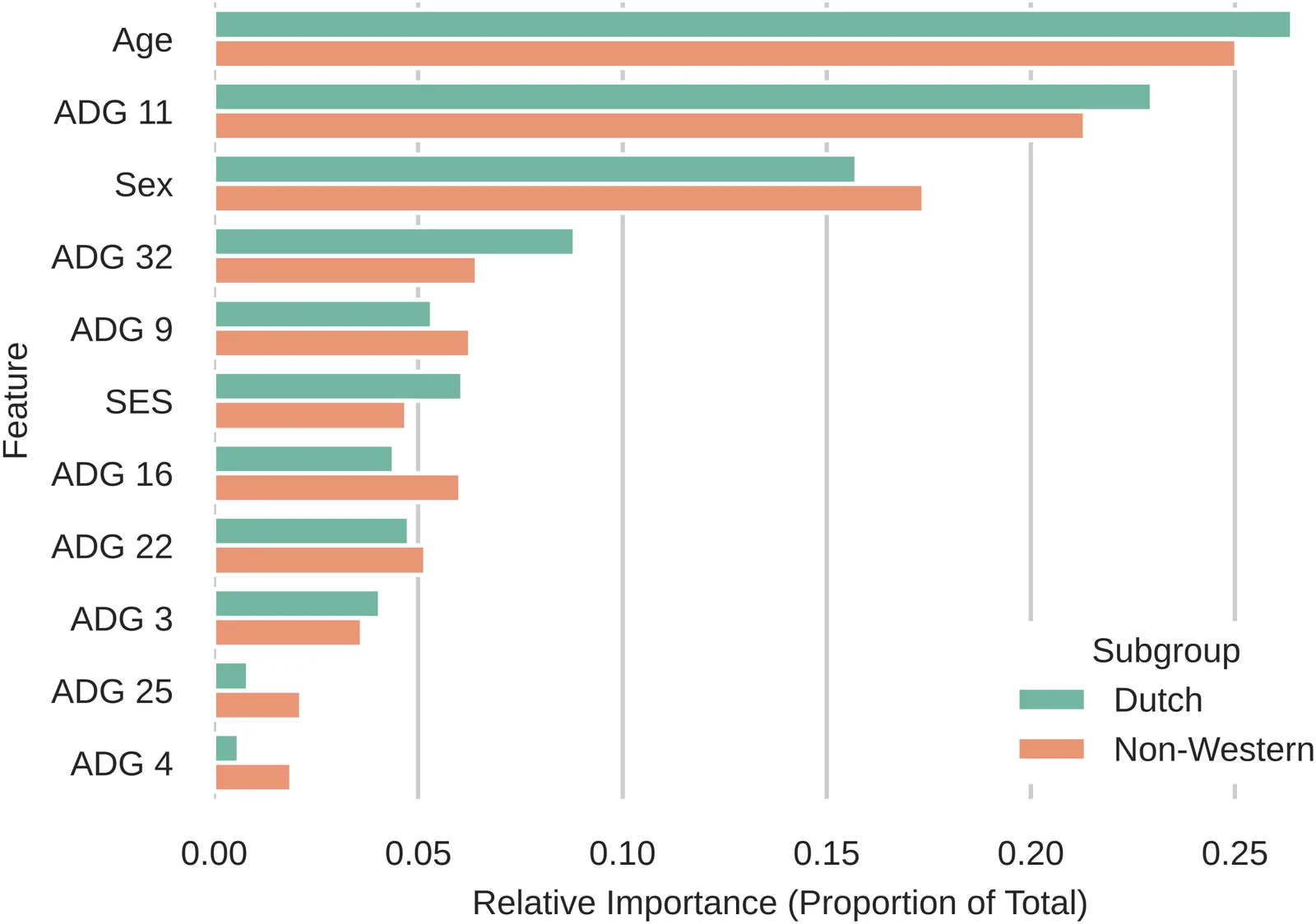
Kernel Shapley Values; Subset of data used, with 1000 positive event (hospitalization) and 1000 negative event (no hospitalization).

When Kernel SV were computed separately for the Dutch and non-Western subgroups using a shared major ADG model, ADG 32 and ADG 11 showed higher mean absolute contributions in the Dutch subgroup (57.3 vs. 37.6 and 148.9 vs. 124.6, respectively; S6 Table). This suggests that these features explained a larger share of the model's predictions in this population, which partly reflects their higher prevalence in the Dutch subgroup (ADG 32: 5.6% vs. 2.6%; ADG 11: 14.6% vs. 11.6%; S2 Table). By contrast, ADG 4 (Time Limited: Major-Primary Infections), ADG9 (Likely to Recur: Progressive), ADG 16 (Chronic Specialty: Unstable Orthopedic) and ADG 25 (Psychosocial: Recurrent or Persistent, Unstable) showed higher SV contributions in the non-Western subgroup.

### Step 6: Compare error attribution features

The Cohort Shapley Values (Fig 6; S7 Table) specifically measured feature contributions to the prediction errors based in only observed combinations in data. Notably, ADG 11 appeared particularly influential in contributing to disparate impact within false negative cases but played a less prominent role in overall residuals. Overall, the FNR tended to be higher in the non-Western group, aligning with higher FNR fairness metrics for that subgroup (Table 2), possibly indicating under-recognition of hospitalization risk among non-Western patients.

**Table 2:** Performance metrics. Model column indicates type of model, where LR stands for Logistic regression. Models were trained on the trainingsdataset of 84,654 patients of which 16% non-western, if not specified otherwise. The balanced dataset consisted of 13,592 Dutch and 13,592 non-western patients. EO: equal odds ratio. FNR: False Negative Rate** Standard deviations across standard metrics were all < 0.02 and are not displayed for clarity. N.A.: Not Applicable.

Model	Test sample	F1 score	Precision	Accuracy	ROC-AUC	EO	FNR
*LR - 32 ADGs*	*Full (28218)*	0.286	0.183	0.684	0.729	0.899	0.335
*LR - 32 ADGs*	*Dutch (23566)*	0.286	0.182	0.678	*See* Fig 4	*n.a.*	0.333
*LR - 32 ADGs*	*Non-western (4652)*	0.290	0.187	0.706	*See* Fig 4	*n.a.*	0.349
*LR - 32 ADGs*	*>= one major ADG (10492)*	0.341	0.233	0.623	0.675	0.878	0.366
*LR - major ADGs*	*Full (28218)*	0.268	0.173	0.684	0.693	0.820	0.421
*LR - major ADGs*	*>= one major ADG (10492)*	0.329	0.223	0.610	0.657	0.775	0.383
*LR - 32 ADGs -* *balanced dataset*	*Full (28218)*	0.285	0.183	0.689	0.729	0.904	0.329
*LR - 32 ADGs-* *balanced dataset*	*Dutch (23566)*	0.285	0.179	0.67	0.674	*n.a.*	0.326
*LR - 32 ADGs -* *balanced dataset*	*Non-western (4652)*	0.288	0.185	0.700	0.68	*n.a.*	0.344
*LR Rx-MG*	*Full (28218)*	0.306	0.204	0.734	0.680	0.971	0.387
*LR Rx-MG*	*Dutch (23566)*	0.309	0.206	0.734	*See* Fig 4	*n.a.*	0.384
*LR Rx-MG*	*Non-western (4652)*	0.294	0.195	0.734	*See* Fig 4	*n.a.*	0.402
*XGBoost - 32 ADGs*	*Full (28218)*	0.164	0.147	0.818	0.709	0.925	0.813
*XGBoost - 32 ADGs*	*Dutch (23566)*	0.167	0.150	0.815	*See* Fig 4	*n.a.*	0.808
*XGBoost - 32 ADGs*	*Non-western (4652)*	0.150	0.139	0.830	*See* Fig 4	*n.a.*	0.837

**Fig 6:**
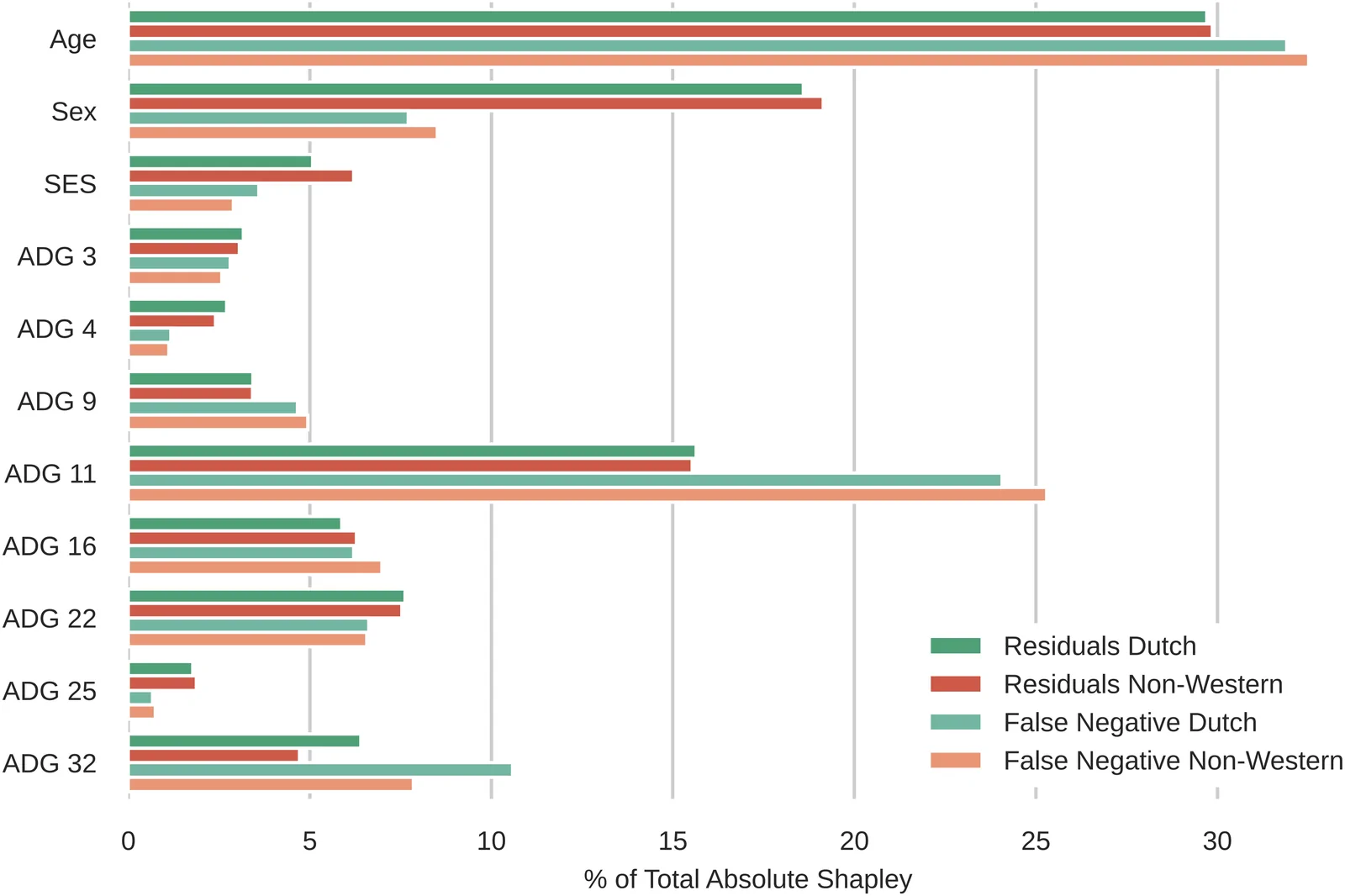
Cohort Shapley Values used to calculate feature contributions to residuals (n = 27,184) and false negative cases. False negatives were analyzed in the subset of individuals with a hospitalization outcome; as this subset contained slightly more Dutch than non-Western individuals, equal groups of 1,235 were sampled.

The lower responsiveness of ADG 32 for residuals and false negatives compared to the Dutch population (Fig 6; S7 Table), further suggests that, when malignancy was diagnosed, it played a different role in shaping prediction errors across subgroups.

### Interpreting differences in feature behavior

To synthesize findings across the three complementary analytical approaches, Fig 7 presents a structured comparison of results for ADG 11 (Chronic Medical: Unstable) and ADG 32 (Malignancy), selected for their prominent role in predictions and divergent patterns. Coefficients are drawn from subgroup-specific 32-ADG models to better contextualize feature behavior within each population's total morbidity profile. Kernel Shapley Values reflect how the *model* translates features into predictions under learned feature dependencies, while Cohort Shapley reflects feature contributions to *observed outcomes* within cohorts of empirically similar patients, enabling comparison between model-implied and observed patterns.

**Fig 7:**
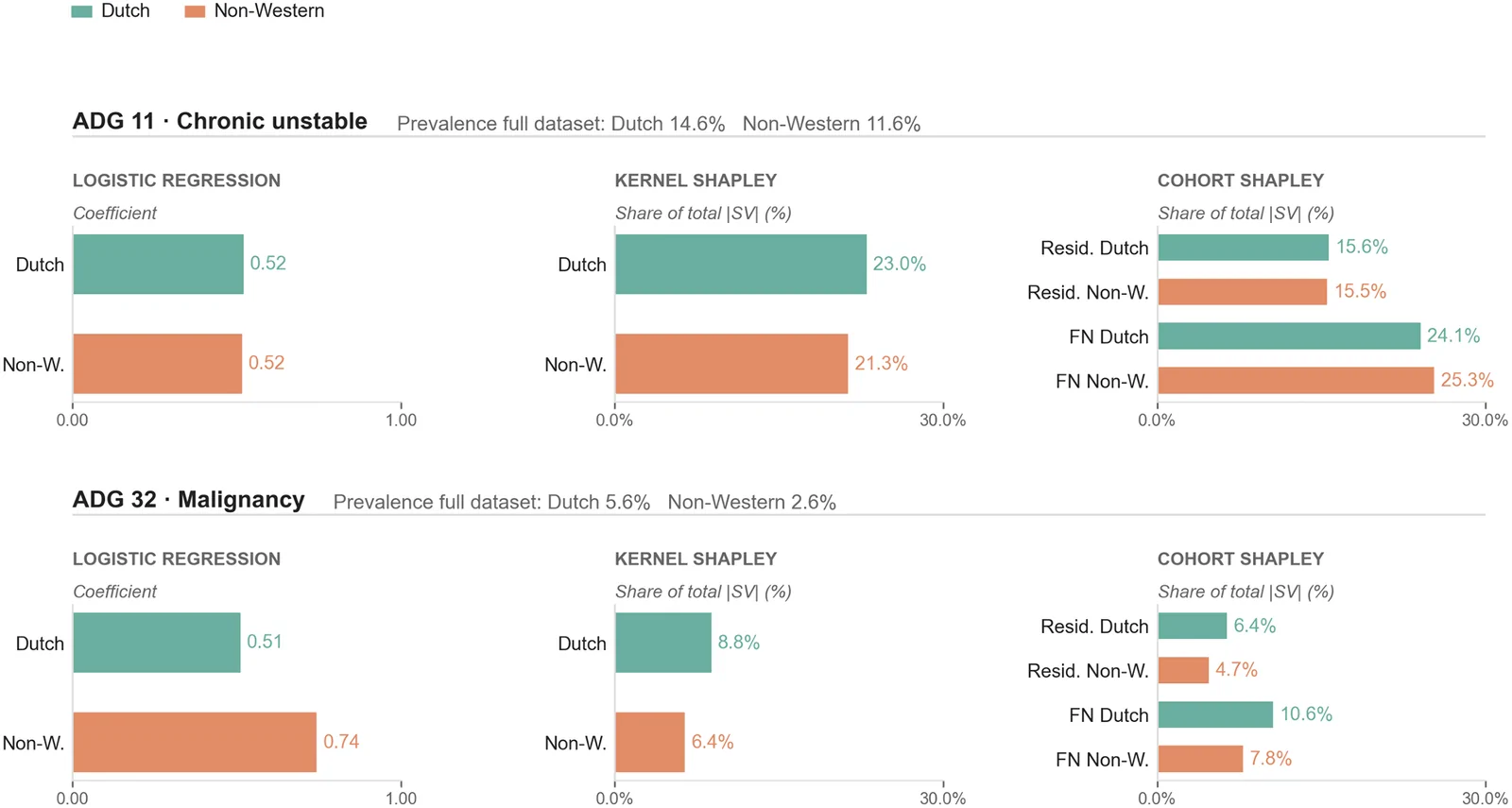
Comparison of feature behavior for ADG 11 (Chronic Medical: Unstable) and ADG 32 (Malignancy) in three analytical approaches. Logistic regression coefficients are from subgroup-specific 32-ADG models. Kernel Shapley values and Cohort Shapley values are shown as each feature's share of total absolute Shapley values for the pooled major ADG model. Cohort Shapley results are presented separately for residuals (Resid.) and false negatives (FN).

ADG 11 and ADG 32 have similar coefficients in the Dutch subgroup, and ADG 11 remains comparable between subgroups (≈0.51). In contrast, ADG 32 has a substantially higher coefficient in the non-Western subgroup model. In the pooled model, this would be expected to lead to an underestimation of hospitalization risk for non-Western patients with ADG 32, and thus a higher contribution of ADG 32 to false negatives in the non-Western group. However, this pattern is not observed in the empirical data of observed hospitalization cases, where Cohort Shapley values for ADG 32 were lower in the non-Western group.

The lower prevalence of ADG 32 in the overall non-Western subgroup likely contributes to this pattern, as fewer patients are exposed to this feature and normalized Shapley Values contributions are correspondingly reduced. Nonetheless, this alone is unlikely to fully explain the observed differences, given the stronger coefficient in this subgroup. In addition, because false negative analyses are restricted to observed hospitalizations, subgroup differences in ADG 32 may also reflect differences in co-morbidity composition of hospitalized cases.

ADG 11 is a heterogeneous category with similar coefficients (Dutch: 0.520; non-Western: 0.515), in contrast to other major ADGs, whose coefficients are generally higher in the non-Western group, except for ADG 25, which contributes minimally. Although correlation analyses show that ADG 11 has no dominant comorbidity association, its heterogeneous nature within the ADG category may obscure subgroup differences, including co-occurrence patterns that the MCA did not capture. A slightly lower prevalence in non-Western patients (11.6% vs. 14.6% in Dutch) further complicates interpretation, as potential underdiagnosis or differences in clinical coding cannot be excluded. An absence of ADG11 is either an absence of disease or an absence of registration. Overall, these findings suggest that ADG 11 may capture different underlying clinical contexts across subgroups, and its heterogenous nature and relatively high contribution of FN cases indicates that further investigation is needed.

## Discussion

Algorithms learn patterns in data. As a result, algorithmic fairness depends on awareness of the (societal) patterns reflected in dataset structures. While performance differences between subgroups may partly reflect variance in outcome prevalence, which is difficult to overcome, systematic bias in the data, such as differences in diagnostic documentation or treatment practices, is potentially correctable when detected, both in the practices that generate the data and, within limits, during modelling. In our study, we systematically examined two subpopulations defined by migration background using our Feature-Level Bias Identification And Sensemaking (FL-BIAS) framework. We demonstrated that, beyond standard performance metrics, valuable insights can be obtained by integrating complementary data science methods. Particularly, we highlighted the added value of using Shapley analyses: Shapley Values can help uncover feature-level bias patterns that would otherwise remain hidden in aggregate metrics. While this particular case study relied on ADGs from the widely used Johns Hopkins ACG system, our fairness diagnostic approach FL-BIAS is broadly applicable to any other structured representations or clinically meaningful subsets of EHR data.

The main strength of our case study was the use of data from Statistics Netherlands, which enabled us to adjust for SES and to create subpopulations of different migration backgrounds. Our developed models using ADG classification appeared to have mild degrees of unfairness, which in this context is defined as a disparity in FNR and EO ratio. Discrepancies in FNR among groups implied that the model underestimates or encounters more difficulty in identifying positive outcomes for a subgroup. When predictive models were trained on pooled data (from Dutch and non-Western combined), the model learned a population-average association between features and outcomes. As a result, it tended to underestimate hospitalization risk for non-Western patients, contributing to higher FNR in this group. Unequal EO ratios entails an uneven distribution of true positives and false positives across different subgroups. The slightly lower EO ratio observed in our study (below one) may reflect several factors, including disparities in the distribution of ADG variables between groups, potential diagnostic differences, and variations in how these features relate to hospitalization outcomes.

While the ADG model performed slightly better overall performance in the non-Western population, a contrasting trend was observed for the Rx-MG model, which is based on medication prescription data. Reducing the ADG model to the eight major ADGs increased unfairness slightly. We further observed that certain ADGs were assigned different weights in the logistic regression model trained on the Dutch dataset as compared to the one trained on the non-Western dataset. The Shapley Values analysis highlighted a different pattern for ADG 32 (Malignancy) and ADG 11 (Chronic Medical: Unstable). While ADG 32 had higher coefficients in the non-Western model, it contributed less to model’s error compared to the Dutch database. This could be potentially explained by the well-documented disparities in screening participation for cancer [49–51], where non-participation can lead to more advanced forms of cancer in the non-Western population at time of diagnosis, consequently establishing stronger associations with hospitalization outcomes. Such a pattern may reflect a form of negative legacy bias [3], where delays in diagnosis become encoded in the data and influence subsequent algorithmic predictions.

As stated in the method section, Kleinberg's impossibility theorem [45] proves that when base rates differ between groups and prediction is imperfect, not all fairness metrics can be met. Oneto and Chiappa [52] further argue that fairness criteria such as Equalized Odds can be misleading when outcomes are affected by unfair causal influence from the sensitive attribute.From our mediation analyses on our dataset, the direct link between migration background and hospitalization appeared not to be statistically significant. However, since variables such as health-care access were unobserved, hospitalization may not accurately represent a patient’s underlying health need. Regarding the ACME analysis, our SES did not include education levels due to high levels of missing data. This exclusion may have reduced the validity of SES as a proxy measure and contributed to the fragility of the ACME estimate, which was nullified at a rho of 0.1. The non-monotonic relationship between SES and hospitalization risk, where Low SES shows a weaker association than Middle SES, may reflect limitations in our SES measure. This is particularly relevant for the interpretation of ADG 32, as SES is independently associated with lower cancer screening rates and variations in cancer type prevalence [53]. Therefore, the unobserved confounding and imperfect measurement of SES limit the extent to which causal conclusions can be drawn. Another limitation for a correct interpretation of our findings is the heterogeneity of the non-Western group. For instance, Arsenijevic et al. [50] reported no difference in knowledge of cervical cancer screening between second-generation non-Western migrants and non-migrant populations after controlling for confounders using propensity score matching. Yet in our study second-generation non-Western migrants are included in our non-Western subgroup, despite their differences in health literacy. This suggests that individuals grouped together may not be comparable in relevant characteristics, making the choice of similarity metrics critical for achieving fairness at individual level, as argued by Dwork et al. [54]. Defining similarity would entail a very granular specification, as second-generation migrants do appear to differ in knowledge of breast and colorectal cancer screenings [50].

Our findings should therefore be viewed as an initial exploratory analysis of the extent of algorithmic bias in the real-world EHR data, serving as an example of how to begin uncovering the root causes of disparate outcomes. The main limitation was its models’ underperformance, which reduced the reliability and interpretive validity of the fairness metrics, feature importance estimates, and Shapley Value analyses. We found that our modelling approach and performance are directly comparable to prior work such as [35], who reported a C-statistic of 0.73 for prospective hospitalization prediction using ACG-based logistic regression models on integrated EHR data at a major US healthcare system. Our model achieves a similar C-Statistic (0.729) despite relying on a more limited primary care EHR dataset. We included a supplement of a literature overview of studies using ACG -based models for a more in-depth comparison (see S8 Table), identifying limitations such as very low event rates and their lack of sensitivity metrics. Reporting Precision-Recall Area Under the Curve (PR-AUC), which is sensitive to outcome prevalence, would have enabled more reliable comparison across studies with low event rates. Most of these studies used multiple data sources, such as prior healthcare utilization and medication prescriptions in addition to diagnosis codes, similar to how the ACG System often is applied in clinical practice. For purposes of our study, we consider it a strength of our study that we explicitly disentangle patterns of medication data (Rx-Mg) and patterns on diagnoses (ADG), even at the cost of some reduction in overall predictive performance. The predictive signal in ADG data more than one year prior to hospitalization is fundamentally limited, although this reflects how the Johns Hopkins ACG system is used for risk stratification in practice. Prior work has shown that a substantial proportion of outcome variability remains unexplained even with the best-performing models [35]. This is further supported by a Norwegian study [32] reporting a higher C-statistic than ours (0.83 vs. 0.729) by using same-year data for hospitalization, thereby reflecting more immediate clinical information. Additionally, incompleteness in EHR data, where one US-based study found that approximately 45% of diagnoses were captured only in claims and missing from EHRs [55], may have further constrained our model sensitivity. Lastly, Haas et al. [35] demonstrated that models with moderate discrimination can still provide clinically useful risk stratification, showing that the highest-risk decile contained a substantially higher rate of hospitalizations compared to the general population (27% top decile vs. 8% baseline). This aligns with the intended use of the ACG system as a tool for population-level risk stratification.

Despite the comparable performance, these limitations affect the interpretation of the feature-level results. In particular, our XGBoost model was underperforming, likely due to the combination of many weakly informative ADG predictors, binary feature sparsity and a lack of strong feature interactions that gradient boosting typically exploits. As shown in Supplement Table 8, ACG-based studies using alternative machine learning algorithms, such as multilayer perceptrons, have only marginally outperformed logistic regression (ΔAUC = 0.0034) [36] or performed worse (ΔAUC = -0.035) [34]. Deep learning architectures specifically designed for tabular data, such as TabNet [56], may have better handled the combination of sparse uninformative features and class imbalance.

Several aspects of our design nevertheless limited the impact of these performance issues. Our use of the major ADG model, with its limited number of predictors, mitigated the risk of the sparse conditioning problem, which affects empirical conditional Shapley estimation in continuous or high-dimensional feature spaces [57]. Moreover, we used multiple analytical approaches and model types to identify any feature-level biases in the underlying data. However, we acknowledge that lower predictive performance may introduce additional noise, potentially affecting the stability of these estimates and the precision of absolute Shapley value estimates. The use of SV remains valid for comparative purposes where relative differences can provide meaningful insights into potential sources of disparity in EHR data, even if the model's overall predictive accuracy is limited. Any observed differences between the two datasets, such as with ADG 11 and ADG 32, may reflect systematic differences in how the model utilizes features and should be used as hypothesis-generating diagnostic signals. Without such diagnostic analyses, fairness interventions risk being based on untested or overly simplistic assumptions about the dataset.

As argued by Corbett-Davies et al. [10], any technical results in the domain of algorithmic fairness must be made relevant to real-world contexts. A multidisciplinary team is needed to uncover underlying data-generating processes, such as variation in clinical recording practices, non-random missingness, or unmeasured variables affecting both health status and healthcare access. Moreover, the use of EHR for observational research is prone to various types of bias [58], which could distort interpretation. Stakeholder input is essential to determine whether observed differences reflect true disparities or spurious associations. Ultimately, algorithmic fairness assessments require a close interplay between in-depth data analysis and human interpretation. Therefore, our diagnostic framework operationalizes principles from Critical Data Studies, which emphasize the non-neutrality of data, by promoting reflexivity on data and facilitating sensemaking at the feature level, through which contextual awareness can emerge [59]. We encourage future work to include additional analyses, for instance by testing different machine learning model architectures, comparing calibration curves as demonstrated by [60] or using bootstrapping for more rigorous estimates as applied by [21] for SV analyses.

## Conclusion

In-depth analyses of databases are key for uncovering the underlying causes of algorithmic bias in datasets and for promoting equity in healthcare delivery. This study introduced the Feature-Level Bias Identification And Sensemaking (FL-BIAS) diagnostic approach for detecting bias at the feature level. We demonstrated the use of FL-BIAS in a case study using Dutch primary care EHR data linked with Statistics Netherlands data, developing models based on ADGs from the Johns Hopkins ACG system. We observed modest disparities in overall model performance but notable differences in the relevance of specific ADG features across subgroups. For example, ADG 32 (Malignancy) had a stronger association with hospitalization among non-Western patients, potentially reflecting more advanced disease state.

These results highlight the need for deeper engagement with the data-generating process, including awareness of potential confounders such as cancer screening participation, when assessing algorithmic fairness. Such disparities might also be addressed through more granular feature engineering, such as splitting the feature for malignancy into severity-based categories (e.g., early-stage vs. advanced malignancy) to better capture clinical heterogeneity between and within subpopulations. Healthcare organizations should implement routine fairness diagnostic approaches and involve multidisciplinary teams to make sense of algorithmic bias patterns and communicate them transparently to users, in the same way that user manuals accompany other medical equipment. FL-BIAS provides a practical framework for this essential work.

## Supporting information

S1 Table Model specifications. Contains detailed model parameters and hyperparameter grid search settings.
(DOCX)

S2 Table Overview prevalence ADG variables for Dutch and non-Western populations. Includes point prevalence differences and p-values from Pearson's Chi-square Test.
(DOCX)

S3 Table Causal Mediation Analysis. Effect measures of the influence of the independent variable (migration background) on the dependent variable (Hospitalization) through the mediation variable (SES). Provides Average Causal Mediated Effect, Average Direct Effect and Rho Value.
(DOCX)

S4 Table Matthews correlation coefficients. Values for Dutch and non-Western logistic regression models, Rx-MG model, and XGBoost model.
(DOCX)

S5 Table Feature influence. Variance Inflation Factor (VIF) indicates the extent of multicollinearity in the dataset. Overview of coefficients of the logistic regression models with 32 ADGs, trained on the full population, Dutch population, and non-Western population.
(DOCX)

S6 Table Kernel Shapley Values. Overview global Shapley values using Kernel Shapley Values to calculate feature contributions to deviations from a baseline prediction (“none”).
(DOCX)

S7 Table Overview global Cohort Shapley Values to calculate feature contributions to residuals and false negative cases. (DOCX)

S8 Table Overview literature ACG-based models. (DOCX)

S1 Fig Correlation heatmap of ADG features. Visualization of correlations between ADG variables.
(TIF)

S1 File Multiple Correspondence Analysis. Sample population characteristics for MCA analysis and Eigenvalues of the first ten dimensions.
(DOCX)
