# Supplementary material for: Refocusing algorithmic fairness on feature-level bias: A diagnostic approach using dutch EHR data
Source: PLOS Digit Health. 2026 Oct 26;5(10):e0001105. doi: 10.1371/journal.pdig.0001105 (PMC13704874; doi:10.1371/journal.pdig.0001105)
Supplement: Supplementary file 1 [file pdig.0001105.s001.docx]

# **Model specifications**

| **Model** | **Training data** | **Feature transformation** | **Parameters** |
| --- | --- | --- | --- |
| *Logistic Regression - 32 ADGs* | 84.654 patients  (71.062 Dutch,  13.592 non-Western) | One-hot encoded variables: Age (binned), SES, Sex | L2 regularization, class weight = balanced |
| *Logistic Regression - 32 ADGs Dutch* | 71.062 Dutch patients | One-hot encoded variables: Age (binned), SES, Sex | L2 regularization, class weight = balanced |
| *Logistic Regression - 32 ADGs Non-Western* | 13.592 non-Western  patients | One-hot encoded variables: Age (binned), SES, Sex | L2 regularization, class weight = balanced |
| *Logistic Regression - major ADGs* | 84.654 patients (\  (71.062 Dutch,  13.592 non-Western) | One-hot encoded variables: Age (binned), SES, Sex | L2 regularization, class weight = balanced |
| *Logistic regression- Rx-MG* | 84.654 patients  (71.062 Dutch,  13.592 non-Western) | One-hot encoded variables: Age (binned), SES, Sex | L2 regularization, class weight = balanced |
| *XGBoost* - 32 ADGs* | 84.654 patients  (71.062 Dutch,  13.592 non-western) | One-hot encoded variables: Age (binned), SES, Sex | nrounds = 300,  max_depth = 10  eta = 0.3  gamma = 0,  colsample_bytree = 0.9  min_child weight=1  subsample = 1  scale_pos_weight = 10  objective= binary:logistic  threshold = 0.5 |

*Overview of model specifications. *XGBoost hyperparameters were optimized using grid search (nrounds: 100–300, max_depth: 6–10, eta: 0.01–0.3, gamma: 0–5, colsample_bytree: 0.3–0.9, min_child_weight: 1–10, subsample: 0.5–1.0), with 5-fold cross-validation via caret::trainControl and F1-score as the optimization metric. Scale_pos_weight was tuned manually using the class ratio as an anchor point (scale_pos_weight = n_negative / n_positive = (1 − 0.097) / 0.097 ≈ 9.3), with final value set to 10.*
