# Supplementary material for: Refocusing algorithmic fairness on feature-level bias: A diagnostic approach using dutch EHR data
Source: PLOS Digit Health. 2026 Oct 26;5(10):e0001105. doi: 10.1371/journal.pdig.0001105 (PMC13704874; doi:10.1371/journal.pdig.0001105)
Supplement: Supplementary file 4 [file pdig.0001105.s004.docx]

# **Matthews Correlation coefficients**

|  | Dutch (23566) | Non western (4652) |
| --- | --- | --- |
| MCC LR-32 ADGs | 0.214 | 0.225 |
| *MCC Parity* | *1* | *1,051* |
| MCC LR Rx-MG | 0.236 | 0.222 |
| *MCC Parity* | *1* | *0.941* |
| MCC XGBoost | 0.170 | 0.185 |
| *MCC Parity* | *1* | *1,090* |

*Matthews Correlation Coefficient (MCC)*
