# Supplementary material for: Refocusing algorithmic fairness on feature-level bias: A diagnostic approach using dutch EHR data
Source: PLOS Digit Health. 2026 Oct 26;5(10):e0001105. doi: 10.1371/journal.pdig.0001105 (PMC13704874; doi:10.1371/journal.pdig.0001105)
Supplement: Supplementary file 8 [file pdig.0001105.s008.docx]

**Overview of ACG-based predictive models in the literature**

ADGs (Aggregated Diagnosis Groups) are diagnosis-level features that represent different dimensions of morbidity and can be used simultaneously as multiple predictors, whereas ACGs (Adjusted Clinical Groups) are patient-level categories derived from combinations of ADGs, together with age and sex, into a single mutually exclusive grouping reflecting overall morbidity burden^[1]^.

Direct AUC comparisons between predictive models are confounded by differences in outcome definition (hospitalization vs. cost vs. mortality vs. complex-patient identification), prediction horizon (same-year vs. prospective), data (primary care only vs. integrated with hospital, medication, or prior-cost data), outcome prevalence (0.9% to 10.1%), and metrics reporting practices. Most studies report AUC as the primary performance metric and do not include population-wide sensitivity, which makes AUC hard to interpret when trained on populations with low event rates.

The table below is not a complete overview as most articles produced several models but a selection of the most relevant models for comparison with our study. We did not include cost-based ACG models, which address a different prediction task.

| **Reference (year, country)** | **Model type** | **Outcome & prevalence** | **C-statistic (AUC)** | **Notes relevant to comparison** |
| --- | --- | --- | --- | --- |
| **Current study (2026, Netherlands)** | Logistic Regression, 32-ADG features (baseline) | Prospective hospitalization ( 9.7%) | 0.729 | - Primary care EHR only - Diagnoses data only ( medication, cost data and prior utilization not included) - Reports MCC, F1, precision, and FNR alongside AUC |
| **Hosar 2024 (Norway)(1)** | Logistic regression, ACG features | Concurrent hospitalization (10.1%) | 0.83 | - Uses same-year input and outcome data - Diagnoses data only (from hospital EHR) |
| **Haas 2013 (USA) (2)** | Logistic regression, ACG features | Prospective hospitalization (8%) | 0.73 | - Integrated system (Mayo Clinic) with inpatient and outpatient data |
| **Girwar 2022 (Netherlands)(3)** | Logistic regression, ADG features | complex patient identification (0.9%) | 0.9 | - Calibration plot indicates overestimation of risk - Absence of sensitivity metrics |
| **Austin 2012 (Canada) (4)** | Logistic regression, ADG features | mortality prediction (3.1%) | 0.84–0.87 | - Data consists exclusively of patients with diabetes - Absence of sensitivity metrics |
| **Lemke 2012 (USA)(5)** | Logistic regression, ACG | Prospective acute care hospitalization (3.2%) | 0.80 | - Outcome excludes childbirth and injury hospitalizations - US commercial insurance population (mean age 35.8, only 4.4% aged 65+) - Includes prior utilization data - Positive predictive value of 0.148 at the 90th-percentile threshold - authors are ACG system developers |
| **Tan 2023 (Singapore) (6)** | Logistic Regression + ML (Random Forest, Neural Net), ACG features | Prospective ≥3 hospital admissions (1.3%) | LR: 0.877  Best ML: 0.842 | - Based on hospital-derived data - Study is evaluating ranking ability (therefore absence of sensitivity metrics) - Targeted at high risk group (minimum of 3 hospitalizations) |
| **Olza 2023 (Spain) (7)** | Logistic regression + ML (Random Forest, Gradient Boost Decision Trees, Neural Net), ACG features | Prospective unplanned hospitalization (5.67%) | LR: 0.7985  best ML (MLP): 0.8019 | - Includes diagnoses and pharmacy data - threshold-specific metrics reported only at fixed high-risk group cutoffs (top 20,000; top 5%; top 10%), not as population-wide sensitivity - recall at top 20,000 (R@20k) was 0.08, meaning the high-risk group captured <10% of actual hospitalizations |

1.	Hosar R, Berntsen GR, Steinsbekk A. Validity of the Johns Hopkins Adjusted Clinical Groups system on the utilisation of healthcare services in Norway: a retrospective cross-sectional study. BMC Health Serv Res. 2024;24(1):1279.

2.	Haas LR, Takahashi PY, Shah ND, Stroebel RJ, Bernard ME, Finnie DM, Naessens JM. Risk-stratification methods for identifying patients for care coordination. The American journal of managed care. 2013;19(9):725-32.

3.	Girwar SM, Verloop JC, Fiocco M, Sutch SP, Numans ME, Bruijnzeels MA. Identifying complex patients using Adjusted Clinical Groups risk stratification tool. Am J Manag Care. 2022;28(4):e140-e5.

4.	Austin PC, Shah BR, Newman A, Anderson GM. Using the Johns Hopkins' Aggregated Diagnosis Groups (ADGs) to predict 1-year mortality in population-based cohorts of patients with diabetes in Ontario, Canada. Diabet Med. 2012;29(9):1134-41.

5.	Lemke KW, Weiner JP, Clark JM. Development and Validation of a Model for Predicting Inpatient Hospitalization. Medical Care. 2012;50(2).

6.	Tan JK, Zhang X, Cheng D, Leong IYO, Wong CS, Tey J, et al. Using the Johns Hopkins ACG Case-Mix System for population segmentation in a hospital-based adult patient population in Singapore. Bmj Open. 2023;13(3):e062786.

7.	Olza A, Millán E, Rodríguez-Álvarez MX. Development and validation of predictive models for unplanned hospitalization in the Basque Country: analyzing the variability of non-deterministic algorithms. BMC Medical Informatics and Decision Making. 2023;23(1):152.

1. Johns Hopkins University. (2022). *The Johns Hopkins ACG® system: Version 13.0 technical reference guide*. Johns Hopkins Bloomberg School of Public Health.
