# Supplementary material for: Refocusing algorithmic fairness on feature-level bias: A diagnostic approach using dutch EHR data
Source: PLOS Digit Health. 2026 Oct 26;5(10):e0001105. doi: 10.1371/journal.pdig.0001105 (PMC13704874; doi:10.1371/journal.pdig.0001105)
Supplement: Supplementary file 10 [file pdig.0001105.s012.docx]

Dear prof. dr. Krasimira Tsaneva-Atanasova,

Thank you very much for the opportunity to revise our manuscript. We are grateful to the reviewers for their constructive feedback, which has helped us significantly improve the clarity and depth of our work. We understood that their biggest concern was the limited model performance. In this revision, we have expanded our discussion of model performance and model selection and added a new supplementary file to incorporate additional ACG-based literature for a better comparison of our models.

For your convenience, we have addressed the comments in the table below. The “Answer” column provides our reasoning, while the “Modifications” column details the corresponding line numbers and specific changes in the revised text. We took the opportunity to make some light language edits in a limited number of places. All changes in the manuscript are highlighted in track changes, as requested.

Should you have any additional comments or concerns, we would be pleased to address them.

Sincerely,

Cathleen S. Parsons

*(On behalf of the authors)*

| **Reviewer 1 Comment** | **Answer** | **Modifications** |
| --- | --- | --- |
| This manuscript proposes and applies a framework to diagnose feature-level biases in electronic health record data. The authors' approach, which goes beyond aggregated fairness metrics, is valuable. Linking EHR data with sociodemographic information from Statistics enables a rich, contextually relevant analysis of disparities. | Thank you for commenting on the value of our work. |  |
| The primary concern lies in the suboptimal performance of the predictive models. Although the authors justify Shapley's analysis for comparative purposes, the models' poor performance casts doubt on the generalizability and robustness of interpretations of equity metrics and trait contributions. Even though the authors acknowledge the models' performance as a significant limitation, they should present a more robust discussion, highlighting which implications of these limitations could affect the conclusions, which should be detailed more explicitly, and which would reinforce the justification for proceeding with the analysis. | We thank the reviewer for this important comment, as it allowed us to reflect on and strengthen our work. We have revised the discussion section to clarify these implications more explicitly.  We acknowledge we haven’t sufficiently addressed the performance of our models. We have now included more literature on ACG-based logistic regression models and added an additional supplementary file 10. We believe our ADG-model performed as expected, as we found that our modelling approach and performance is highly comparable to prior work such as Haas et al. (1), which achieved similar C-statistics of 0.73. Furthermore, we have substantially rewritten our discussion, addressing the fundamental limit of EHR data for prediction of prospective hospitalization, while emphasizing this is the clinical use of ACG system, one of the world’s most widely used population health stratification tools.  Secondly, we now explicitly acknowledge that moderate predictive performance increases the risk of capturing noise. However, we would like to point out that in healthcare data variability is often not purely random but likely reflects systematic differences in data collection, coding practices, and care processes. Our model based on medication data revealed lesser degree of algorithmic bias (RX-MG model). Furthermore, while moderate model performance may affect the precision of absolute Shapley value estimates, the relative differences in feature contributions between subgroups are more robust, as they reflect systematic patterns in the underlying data structure that persist across multiple analytical approaches and model types. We have added a section ‘Interpreting differences in feature behavior’ to emphasize more clearly that these findings should be interpreted as hypothesis-generating as opposed to causal, in line with our sociotechnical perspective.  Finally, we would like to clarify that the primary aim of this study is to encourage more critical pre-assessment of potential biases before applying mitigation strategies in practice. We believe that our analyses are sufficiently robust to highlight the importance of this step, while acknowledging the limitations imposed by model performance for any causal conclusions. | Added: Supplementary file 10  Line number 605: Nevertheless, we found that our modelling approach and performance are directly comparable to prior work such as (1) , who reported a C-statistic of 0.73 for prospective hospitalization prediction using ACG-based logistic regression models on integrated EHR data at a major US healthcare system. Our model achieves a similar C-Statistic (0.729) despite relying on a more limited primary care EHR dataset. We included a supplement of a literature overview of studies using ACG -based models for a more in-depth comparison (see S10 Table), identifying limitations such as very low event rates and their lack of sensitivity metrics. Reporting Precision-Recall Area Under the Curve (PR-AUC), which is sensitive to outcome prevalence, would have enabled more reliable comparison across studies with low event rates. Most of these studies used multiple data sources, such as prior healthcare utilization and medication prescriptions in addition to diagnosis codes, similar to how the ACG System often is applied in clinical practice. For purposes of our study, we consider it a strength of our study that we explicitly disentangle patterns of medication data (Rx-Mg) and patterns on diagnoses (ADG), even at the cost of some reduction in overall predictive performance. The predictive signal in ADG data more than one year prior to hospitalization is fundamentally limited, although this reflects how the Johns Hopkins ACG system is used for risk stratification in practice. Prior work has shown that a substantial proportion of outcome variability remains unexplained even with the best-performing models (1). This is further supported by a Norwegian study (2) reporting a higher C-statistic than ours (0.83 vs. 0.729) by using same-year data for hospitalization, thereby reflecting more immediate clinical information. Additionally, incompleteness in EHR data, where one US-based study found that approximately 45% of diagnoses were captured only in claims and missing from EHRs (3), may have further constrained our model sensitivity. Lastly, Haas et al. (1) demonstrated that models with moderate discrimination can still provide clinically useful risk stratification, showing that the highest-risk decile contained a substantially higher rate of hospitalizations compared to the general population (27% top decile vs. 8% baseline). This aligns with the intended use of the ACG system as a tool for population-level risk stratification.  Added line number 486: section “Interpreting differences in feature behavior”  Line number 658: Moreover, we used multiple analytical approaches and model types to identify any feature-level biases in the underlying data. However, we acknowledge that lower predictive performance may introduce additional noise, potentially affecting the stability of these estimates and the precision of absolute Shapley value estimates. |
| The authors should provide a clear explanation of the criteria used for choosing the specific models in this study. | We agree that the criteria for model selection were not sufficiently explicit. We have added our reasoning to the Methods section to clarify these choices and included the grid search parameters for the XGBoost model in supplementary file 1. | Line number 251: Logistic regression is a commonly used modelling approach in ACG-based research (1, 2, 4-8), including studies conducted by the Johns Hopkins ACG developers. To account for any non-linear relationships and interactions between the features, we compared the logistic regression model to a XGBoost model, since gradient-boosted trees are among the best-performing methods for tabular prediction tasks (9). |
| Furthermore, the heterogeneity of the non-Western group is a limitation that warrants further discussion, given the nature of the study. The non-Western classification groups are highly heterogeneous. This limitation warrants a more in-depth discussion of how this simplification may mask nuances within subgroups. | We thank the reviewer for raising this important point and agree that the heterogeneity of the non-Western group calls for a more explicit discussion.  In our study, this heterogeneity arises at two levels: between countries of origin and within country groups. To partially address between-country heterogeneity, we applied a minimum sample size threshold (n ≥ 500), resulting in the inclusion of 10 country-of-origin groups: Morocco, Suriname, Iran, Iraq, Turkey, Indonesia, the Dutch Antilles, Dutch Indies, Afghanistan, and the former Soviet Union (which does not include Eastern Bloc countries such as Poland, Hungary, or Romania). Although this is a heterogeneous group, they share the experience of being a minority population interacting with the Dutch health care system which is relevant for our fairness analysis. We have added this to our method section. We did not conduct analyses at the individual country level, as further disaggregation would result in small subgroup sizes and furthermore, we aimed to avoid unnecessary stigmatisation of differences in health status of specific subpopulations.  For the second type of heterogeneity within country-of-origin categories themselves, we acknowledge we hadn’t addressed that sufficiently. We now address that this classification is considered controversial due to the heterogeneity (10), particularly since it includes being allocated to a category due to the parent’s birth country.  Finally, in response to another reviewer’s comment, we have expanded the discussion to distinguish between group-level and individual-level fairness, acknowledging that aggregated categories may obscure important variation at the individual level for cancer screening health literacy. | Line number 197: For migration background, a dataset was used that assigns each subject in the Netherlands to either their own country of birth or, if born in the Netherlands, to the country of birth of their non-Dutch parent (with the mother’s country used by default if both parents are non-Dutch). Although this classification has been subject to public debate (10, 11), it reflects the categorization used by Statistics Netherlands in 2016, on which the linked data are based.  Line number 591: Another limitation for a correct interpretation of our findings is the heterogeneity of the non-Western group. For instance, Arsenijevic et al. (12) reported no difference in knowledge of cervical cancer screening between second-generation non-Western migrants and non-migrant populations after controlling for confounders using propensity score matching. Yet in our study second-generation non-Western migrants are included in our non-Western subgroup, despite their differences in health literacy. This suggests that individuals grouped together may not be comparable in relevant characteristics, making the choice of similarity metrics critical for achieving fairness at individual level, as argued by Dwork et al. (13). Defining similarity would entail a very granular specification, as second-generation migrants do appear to differ in knowledge of breast and colorectal cancer screenings (12). |
| The unobserved confounding variables may weaken the mediation analysis. Furthermore, the sensitivity of socioeconomic status (SES) as an indicator of educational needs requires further explanation, with emphasis on how these factors affect the validity of causal conclusions. | Thank you for highlighting this important methodological point, which we haven’t sufficiently addressed.  As described in the Methods section, data from Statistics Netherlands on education and occupation were not included in our SES measure due to substantial missingness (30%). This omission has reduced the validity of SES as a variable as we now only based it on household income and wealth. For instance, education and occupation are closely linked to health literacy. This limitation is particularly relevant when interpreting ADG 32, as both cancer incidence and screening uptake are known to vary by SES  (12, 14, 15). Therefore, we agree we should more explicitly caution against making causal interpretations regarding the role of migration background in observed differences related to ADG 32, as we have unobserved confounding of educational differences and other socioeconomic factors. We have revised our discussion to address this more explicitly. | Line number 585: This exclusion may have reduced the validity of SES as a proxy measure and contributed to the fragility of the ACME estimate, which was nullified at a rho of 0.1. The non-monotonic relationship between SES and hospitalization risk, where Low SES shows a weaker association than Middle SES, may reflect limitations in our SES measure. This is particularly relevant for the interpretation of ADG 32, as SES is independently associated with lower cancer screening rates and variations in cancer type prevalence (14). Therefore, the unobserved confounding and imperfect measurement of SES limit the extent to which causal conclusions can be drawn. |

| **Reviewer 2 Comment** | **Answer** | **Modifications** |
| --- | --- | --- |
| The paper explores how patient migration background data corrected for SES affect predictions for hospital admissions. It makes the strong point that understanding the underlying context and the data is crucial for understanding algorithmic fairness, and it may not even be possible to mitigate all of the fairness challenges. The paper uses standard data science and machine learning techniques in sensible ways to understand both the data dependencies and biases, and the fairness issues in the predictive methodologies. The analysis is overall convincing. | Thank you for the compliments |  |
| The predictive accuracies of the logistic regression and the XGBOOST based techniques appear to be rather low. Clearly, the demographic fairness question becomes meaningful only when there is high utility in prediction. Would a better model (may be a neural network) not only predict better, but may also uncover the data dependencies and proxies better and hence be able to account for them? Would the interpretations in the paper change in such a case? Or is it the case that the low predictive accuracy is due to omitted variables and the main conclusions will remain the same irrespective of the model? | We thank the reviewer for this important question.  Regarding why predictive accuracy is limited: The predictive signal in diagnostic data recorded one year prior to hospitalization is fundamentally limited and this is a property of the prediction task with limited clinical information, not the model, which we now elaborate on explicitly in our discussion. Our AUC of 0.729 is directly comparable to Haas et al. (0.73), who used richer integrated data from a major US healthcare system, but lower than the AUC of 0.83 of Hosar et al. (2) whose higher performance can be explained by the same-year prediction task (consequently they describe their models as classification not prediction). We have now added an additional supplement where we compare our work with other ACG-based research. The Johns Hopkins ACG system is deployed in clinical practice worldwide under similar conditions. Evaluating fairness under real-world deployment conditions, including their limitations could be considered as a strength of our study design. Lastly, existing literature on ACG-based models shows that more complex architectures provide minimal gains: multilayer perceptrons improve AUC by only 0.0034 over logistic regression (7), and in one study performed slightly worse(6). The sparse binary nature of ADG features and limited feature interactions, confirmed by our VIF and correlation analyses, likely explains this ceiling.  Regarding whether a better model would change our conclusions: although we acknowledge that moderate predictive performance increases the risk of capturing noise, we also highlight that feature-level bias patterns reflect systematic differences in how features relate to outcomes across subgroups and these patterns are more likely to reflect properties of the data structure than model architecture, as evidenced by their persistence across multiple analytical approaches (e.g., ADG logistic regression versus Rx-MG logistic regression, XGBoost versus log reg, Kernel SV, Cohort Shapley ). We are therefore confident enough that the observed differences in ADG behavior reflect genuine data structure patterns. | Added: Supplementary file 10  Line number 605: Nevertheless, we found that our modelling approach and performance are directly comparable to prior work such as (1) , who reported a C-statistic of 0.73 for prospective hospitalization prediction using ACG-based logistic regression models on integrated EHR data at a major US healthcare system. Our model achieves a similar C-Statistic (0.729) despite relying on a more limited primary care EHR dataset. We included a supplement of a literature overview of studies using ACG -based models for a more in-depth comparison (see S10 Table), identifying limitations such as very low event rates and their lack of sensitivity metrics. Reporting Precision-Recall Area Under the Curve (PR-AUC), which is sensitive to outcome prevalence, would have enabled more reliable comparison across studies with low event rates. Most of these studies used multiple data sources, such as prior healthcare utilization and medication prescriptions in addition to diagnosis codes, similar to how the ACG System often is applied in clinical practice. For purposes of our study, we consider it a strength of our study that we explicitly disentangle patterns of medication data (Rx-Mg) and patterns on diagnoses (ADG), even at the cost of some reduction in overall predictive performance. The predictive signal in ADG data more than one year prior to hospitalization is fundamentally limited, although this reflects how the Johns Hopkins ACG system is used for risk stratification in practice. Prior work has shown that a substantial proportion of outcome variability remains unexplained even with the best-performing models (1). This is further supported by a Norwegian study (2) reporting a higher C-statistic than ours (0.83 vs. 0.729) by using same-year data for hospitalization, thereby reflecting more immediate clinical information. Additionally, incompleteness in EHR data, where one US-based study found that approximately 45% of diagnoses were captured only in claims and missing from EHRs (3), may have further constrained our model sensitivity. Lastly, Haas et al. (1) demonstrated that models with moderate discrimination can still provide clinically useful risk stratification, showing that the highest-risk decile contained a substantially higher rate of hospitalizations compared to the general population (27% top decile vs. 8% baseline). This aligns with the intended use of the ACG system as a tool for population-level risk stratification.    Line number 636: In particular, our XGBoost model was underperforming, likely due to the combination of many weakly informative ADG predictors, binary feature sparsity and a lack of strong feature interactions that gradient boosting typically exploits. As shown in Supplement Table 10, ACG-based studies using alternative machine learning algorithms, such as multilayer perceptrons, have only marginally outperformed logistic regression (ΔAUC = 0.0034) (7) or performed worse (ΔAUC = -0.035) (6). |
| What explains that different ADGs are significant predictors for different groups? | We thank the reviewer for this question, as it touches on the core contribution of our paper. We might not have interpreted our findings enough in the first version and therefore have added a dedicated section and figure (figure 7) to the manuscript.  Differences in which ADGs are significant predictors across groups likely reflect three factors. First, genuine differences in underlying disease burden, where subgroups differ in the prevalence and severity of conditions. We hypothesize this for ADG 32. Second, group-specific variation in clinical documentation practices within EHR data, where the same condition may be recorded differently across populations. This might be the case for ADG11, although the data alone is not sufficient for any conclusions. Third, prevalence-driven statistical effects on feature importance estimates, where a feature's contribution to model predictions is partly a function of how frequently it appears in the data. This is the reason why FL-BIAS is a structured approach that includes EDA.  Each of these factors implies a different mitigation strategy. A bias rooted in disease burden requires a different response than one rooted in documentation practices or statistics. This is precisely why pre-assessment at the feature level, before applying mitigation strategies, forms the central argument of our paper. | Added line number 486: section“Interpreting differences in featurebehavior” |
| [32] not only suggests that fairness can be defined in multiple ways, but also that inherent trade offs between individual fairness (calibration) and group fairness are inevitable in real-world data. The paper does not comment on this trade off at all. It will be good if some insights can be included. | This is indeed very relevant to our paper, as it discusses the influence that a different base rate can have. We have expanded our discussion. | Line number 575: As stated in the method section, Kleinberg's impossibility theorem (16) proves that when base rates differ between groups and prediction is imperfect, not all fairness metrics can be met. Oneto and Chiappa (17) further argue that fairness criteria such as Equalized Odds can be misleading when outcomes are affected by unfair causal influence from the sensitive attribute.From our mediation analyses on our dataset, the direct link between migration background and hospitalization appeared not to be statistically significant. However, since variables such as health-care access were unobserved, hospitalization may not accurately represent a patient’s underlying health need. |
| The paper should also cite and discuss "Fairness through Awareness'' by Dwork et al. | Thank you for bringing this to our attention, this is indeed relevant and we have now included Dwork’s paper in our discussion | Line number 591: Another limitation for a correct interpretation of our findings is the heterogeneity of the non-Western group. For instance, Arsenijevic et al. (12) reported no difference in knowledge of cervical cancer screening between second-generation non-Western migrants and non-migrant populations after controlling for confounders using propensity score matching. Yet in our study second-generation non-Western migrants are included in our non-Western subgroup, despite their differences in health literacy. This suggests that individuals grouped together may not be comparable in relevant characteristics, making the choice of similarity metrics critical for achieving fairness at individual level, as argued by Dwork et al. (13). Defining similarity would entail a very granular specification, as second-generation migrants do appear to differ in knowledge of breast and colorectal cancer screenings (12). |

| **Reviewer 3 Comment** | **Answer** | **Modifications** |
| --- | --- | --- |
| This paper is interesting and very complete | Thank you for the kind words. |  |
| I wonder how much the aggregation of diagnoses into ADGs might influence the fairness results. Since ADGs combine conditions with potentially different severity and risk profiles, subgroup differences in ADG coefficients or Shapley values could reflect underlying severity or stage differences rather than true feature-level bias. Do you think using more granular diagnosis codes would change the subgroup fairness patterns you observed? | We thank the reviewer for this insightful observation, which directly supports our interpretation of the ADG 32 findings.  You are right to point out that ADGs aggregate conditions with potentially different severity profiles. The higher coefficient for ADG 32 in the non-Western model likely does reflect underlying severity differences. This is not a limitation of our analysis but it is the finding. The ADG classification system, by aggregating all malignancies into a single binary feature, obscures this severity gradient and our FL-BIAS analysis makes it visible. This is an important finding as this is how the Johns Hopkins ACG system codes diagnoses in clinical practice worldwide.  Your suggestion of using more granular diagnosis codes is a direction for future work, and we explicitly recommend this in our conclusion: splitting ADG 32 into severity-based subcategories such as early-stage versus advanced malignancy. |  |

1.	Haas LR, Takahashi PY, Shah ND, Stroebel RJ, Bernard ME, Finnie DM, Naessens JM. Risk-stratification methods for identifying patients for care coordination. The American journal of managed care. 2013;19(9):725-32.

2.	Hosar R, Berntsen GR, Steinsbekk A. Validity of the Johns Hopkins Adjusted Clinical Groups system on the utilisation of healthcare services in Norway: a retrospective cross-sectional study. BMC Health Serv Res. 2024;24(1):1279.

3.	Xiong MC, Pittell H, Kitchen C, Lasser EC, Kharrazi H. Synergy of diagnosis coding between administrative claims and electronic health records of large patient populations across multiple healthcare organizations. JAMIA Open. 2026;9(2).

4.	Girwar SM, Verloop JC, Fiocco M, Sutch SP, Numans ME, Bruijnzeels MA. Identifying complex patients using Adjusted Clinical Groups risk stratification tool. Am J Manag Care. 2022;28(4):e140-e5.

5.	Austin PC, Shah BR, Newman A, Anderson GM. Using the Johns Hopkins' Aggregated Diagnosis Groups (ADGs) to predict 1-year mortality in population-based cohorts of patients with diabetes in Ontario, Canada. Diabet Med. 2012;29(9):1134-41.

6.	Tan JK, Zhang X, Cheng D, Leong IYO, Wong CS, Tey J, et al. Using the Johns Hopkins ACG Case-Mix System for population segmentation in a hospital-based adult patient population in Singapore. Bmj Open. 2023;13(3):e062786.

7.	Olza A, Millán E, Rodríguez-Álvarez MX. Development and validation of predictive models for unplanned hospitalization in the Basque Country: analyzing the variability of non-deterministic algorithms. BMC Medical Informatics and Decision Making. 2023;23(1):152.

8.	Orueta JF, Nuno-Solinis R, Mateos M, Vergara I, Grandes G, Esnaola S. Predictive risk modelling in the Spanish population: a cross-sectional study. BMC Health Serv Res. 2013;13:269.

9.	Grinsztajn L, Oyallon E, Varoquaux G. Why do tree-based models still outperform deep learning on typical tabular data? In: Koyejo S, Mohamed S, Agarwal A, Belgrave D, Cho K, Oh A, editors.2022. p. 507--20.

10.	Voogt F. Waarom kijkt CBS nog naar het geboorteland van ouders? 'Het doet geen recht aan de diversiteit binnen de groepen' [Newspaper]. NRC; 2025 [cited 2026 April 11]. 22 July:[Available from: <https://www.nrc.nl/nieuws/2025/07/22/waarom-kijkt-cbs-nog-naar-het-geboorteland-van-ouders-het-doet-geen-recht-aan-de-diversiteit-binnen-de-groepen-2-a4900967>.

11.	Statistics Netherlands. Nieuwe indeling bevolking naar herkomst: Vervanging van indeling naar migratieachtergrond en westers/niet-westers 2022 [cited 2026 April 21]. Available from: <https://www.cbs.nl/nl-nl/longread/statistische-trends/2022/nieuwe-indeling-bevolking-naar-herkomst>.

12.	Arsenijevic DJ, Seibel DV. Do immigrants know less than natives about cancer screening tests? – the case of Netherlands. Journal of Migration and Health. 2024;10:100258.

13.	Dwork C, Hardt M, Pitassi T, Reingold O, Zemel R. Fairness through awareness. Proceedings of the 3rd Innovations in Theoretical Computer Science Conference; Cambridge, Massachusetts: Association for Computing Machinery; 2012. p. 214–26.

14.	Integraal Kankercentrum Nederland (IKNL). Kanker in Nederland: sociaaleconomische verschillen. Deel I: verschillen vóór en rondom diagnose [Available from: <https://iknl.nl/kanker-in-nederland-ses-rapport-1>.

15.	Bongaerts THG, Büchner FL, Middelkoop BJC, Guicherit OR, Numans ME. Determinants of (non-)attendance at the Dutch cancer screening programmes: A systematic review. Journal of Medical Screening. 2020;27(3):121-9.

16.	Kleinberg J, Mullainathan S, Raghavan M. Inherent trade-offs in the fair determination of risk scores. arXiv preprint arXiv:160905807. 2016.

17.	Oneto L, Chiappa S. Fairness in Machine Learning. In: Oneto L, Navarin N, Sperduti A, Anguita D, editors. Recent Trends in Learning From Data: Tutorials from the INNS Big Data and Deep Learning Conference (INNSBDDL2019). Cham: Springer International Publishing; 2020. p. 155-96.
