# Supplementary material for: Refocusing algorithmic fairness on feature-level bias: A diagnostic approach using dutch EHR data
Source: PLOS Digit Health. 2026 Oct 26;5(10):e0001105. doi: 10.1371/journal.pdig.0001105 (PMC13704874; doi:10.1371/journal.pdig.0001105)
Supplement: Supplementary file 11 [file pdig.0001105.s013.docx]

Dear prof. dr. Krasimira Tsaneva-Atanasova,

Thank you for the opportunity to revise our manuscript. We agree with Reviewer #2’s central point that the main value of our proposed method is diagnostic and that several phrases in the previous manuscript version still emphasized bias mitigation, which understated this point. In this revision we have made the diagnostic positioning of FL-BIAS more consistent throughout the abstract, discussion, and conclusion.

As before, we address the comments point by point in the table below. All changes are highlighted in track changes. None of the revisions required new analyses, as the reviewer's comments concern the framing and interpretation of our findings.

Sincerely,

Cathleen Parsons , on behalf of all co-authors

# Point-by-point response to Reviewer #2

| **Reviewer #2 Comment** | **Answer** | **Modifications** |
| --- | --- | --- |
| 1) the low predictive accuracy casts doubt on the bias inferences about the data variables. That the predictive accuracy matches the state of the art does not take away the problem | We agree that low predictive accuracy limits the strength of inferences drawn from feature-level results. We had chosen to use a real case study, ACG variables on General Practitioners data, as it interestingly exposes potential sources of bias in real, commonly used data, which is ultimately the main interest.  We have revised the discussion to state explicitly that, given the moderate predictive performance, all feature-level results should be interpreted as hypothesis-generating diagnostic signals about the underlying data and can be considered indicators of where further multidisciplinary investigation is needed. We have also removed wording that overstated the certainty of these findings (for instance change ‘genuine’ differences into ‘systematic’ differences). | Line 578: removed “Nevertheless”  Line 605: added “Despite the comparable performance, these limitations affect the interpretation of the feature-level results. “  Line 614: replaced “ Despite the limitations in model performance, we argue that our case study reflects the kinds of imperfect conditions found in real-world data upon which predictive models are often developed.” Byb “Several aspects of our design nevertheless limited the impact of these performance issues.”  Line 626: Old “Any observed differences between the two datasets, such as with ADG 11 and ADG 32, reflect genuine differences in how the model utilizes features”  New: “Any observed differences between the two datasets, such as with ADG 11 and ADG 32, may reflect systematic differences in how the model utilizes features and should be used as hypothesis-generating diagnostic signals.“ |
| 2) possibilities of omitted variables and unobserved confounding make the inferences and interpretations somewhat uncertain | We agree. Our sensitivity analysis showed that the estimated ACME was nullified at a correlation of ρ = 0.1 between the error terms, and our SES measure excluded education and occupation due to high levels of missing data. We discuss the implications of this in our discussion. To emphasize this important point even more, we have now added this limitation in line 167. We also more explicitly say that the role of FL-BIAS is to generate hypothesis-generating diagnostic signals that must subsequently be examined by multidisciplinary teams with knowledge of the data-generating process. | Line 167: added “after consideration of omitted variables, unobserved confounding, and the social and medical processes that generate the data. “  Line 626: Old “Any observed differences between the two datasets, such as with ADG 11 and ADG 32, reflect genuine differences in how the model utilizes features”  New: “Any observed differences between the two datasets, such as with ADG 11 and ADG 32, may reflect systematic differences in how the model utilizes features and should be used as hypothesis-generating diagnostic signals.“ |
| 3) most importantly, if there is bias in the underlying data then it is in general impossible to mitigate by negating their effects in predictive machine learning systems. This necessitates making normative decisions about fairness criteria explicit and publicly scrutinised; and paying more attention to the social and medical processes that generate the data in the first place. In that sense the main value of the proposed method is as a diagnostic tool rather than as a predictive tool that mitigates the biases. This needs to be adequately highlighted. | We fully agree, and that FL-BIAS is a diagnostic tool is the central message of our paper, as reflected in its title. That fairness issues can originate in historical data biases is illustrated, for example, by the well-known article by Obermeyer et al 2019 and we agree with the importance of paying more attention to the social and medical processes. In our discussion we argue that technical fairness results must be made relevant to real-world contexts and that a multidisciplinary team is needed to uncover the underlying data-generating processes such as variation in clinical recording practices. We accept, however, that this argument was undermined by mitigation-oriented framing elsewhere in the manuscript, and we have now removed that framing so the diagnostic positioning is consistent throughout. | Line 41: old “Fairness in healthcare AI must be approached as a socio-technical challenge, requiring multidisciplinary collaboration to uncover root causes and guide effective mitigation strategies.  New “Fairness in healthcare algorithms must be approached as a socio-technical challenge, requiring multidisciplinary collaboration to uncover root causes in the processes that generate the data.”  Line 508: added “, both in the practices that generate the data and, within limits, during modelling.”  Line 626: Old “Any observed differences between the two datasets, such as with ADG 11 and ADG 32, reflect genuine differences in how the model utilizes features”  New: “Any observed differences between the two datasets, such as with ADG 11 and ADG 32, may reflect systematic differences in how the model utilizes features and should be used as hypothesis-generating diagnostic signals.“  Line 664: removed “, supporting the development of appropriate mitigation strategies and targeted interventions to reduce health disparities. “ |

**Reference**

Obermeyer, Z., Powers, B., Vogeli, C., & Mullainathan, S. (2019). Dissecting racial bias in an algorithm used to manage the health of populations. *Science*, *366*(6464), 447-453.
