# Supplementary material for: Refocusing algorithmic fairness on feature-level bias: A diagnostic approach using dutch EHR data
Source: PLOS Digit Health. 2026 Oct 26;5(10):e0001105. doi: 10.1371/journal.pdig.0001105 (PMC13704874; doi:10.1371/journal.pdig.0001105)
Supplement: Peer review history

# Peer review: Decision Letter 0

**DOI:** 10.1371/journal.pdig.0001105.r001

**Authors:** Krasimira Tsaneva-Atanasova, Mengyu Wang

6 Apr 2026

Response to ReviewersRevised Manuscript with Track ChangesManuscript**Journal Requirements:** **Additional Editor Comments (if provided):** **Reviewers' Comments:**

**Comments to the Author**

1. Does this manuscript meet PLOS Digital Health’s publication criteria?

Reviewer #1: Yes

Reviewer #2: Yes

Reviewer #3: No

**********

2. Has the statistical analysis been performed appropriately and rigorously?

Reviewer #1: Yes

Reviewer #2: Yes

Reviewer #3: Yes

**********

3. Have the authors made all data underlying the findings in their manuscript fully available (please refer to the Data Availability Statement at the start of the manuscript PDF file)?

The PLOS Data policy

Reviewer #1: Yes

Reviewer #2: Yes

Reviewer #3: Yes

**********

4. Is the manuscript presented in an intelligible fashion and written in standard English?

Reviewer #1: Yes

Reviewer #2: Yes

Reviewer #3: Yes

**********

Reviewer #1: This manuscript proposes and applies a framework to diagnose feature-level biases in electronic health record data. The authors' approach, which goes beyond aggregated fairness metrics, is valuable. Linking EHR data with sociodemographic information from Statistics enables a rich, contextually relevant analysis of disparities.

However, some issues require further review and investigation:

1) The primary concern lies in the suboptimal performance of the predictive models. Although the authors justify Shapley's analysis for comparative purposes, the models' poor performance casts doubt on the generalizability and robustness of interpretations of equity metrics and trait contributions. Even though the authors acknowledge the models' performance as a significant limitation, they should present a more robust discussion, highlighting which implications of these limitations could affect the conclusions, which should be detailed more explicitly, and which would reinforce the justification for proceeding with the analysis.

2) The authors should provide a clear explanation of the criteria used for choosing the specific models in this study.

3) Furthermore, the heterogeneity of the non-Western group is a limitation that warrants further discussion, given the nature of the study. The non-Western classification groups are highly heterogeneous. This limitation warrants a more in-depth discussion of how this simplification may mask nuances within subgroups.

4) The unobserved confounding variables may weaken the mediation analysis. Furthermore, the sensitivity of socioeconomic status (SES) as an indicator of educational needs requires further explanation, with emphasis on how these factors affect the validity of causal conclusions.

Reviewer #2: The paper explores how patient migration background data corrected for SES affect predictions for hospital admissions. It makes the strong point that understanding the underlying context and the data is crucial for understanding algorithmic fairness, and it may not even be possible to mitigate all of the fairness challenges. The paper uses standard data science and machine learning techniques in sensible ways to understand both the data dependencies and biases, and the fairness issues in the predictive methodologies. The analysis is overall convincing. I have the following minor observations:

1. The predictive accuracies of the logistic regression and the XGBOOST based techniques appear to be rather low. Clearly, the demographic fairness question becomes meaningful only when there is high utility in prediction. Would a better model (may be a neural network) not only predict better, but may also uncover the data dependencies and proxies better and hence be able to account for them? Would the interpretations in the paper change in such a case? Or is it the case that the low predictive accuracy is due to omitted variables and the main conclusions will remain the same irrespective of the model?

2. What explains that different ADGs are significant predictors for different groups?

3. [32] not only suggests that fairness can be defined in multiple ways, but also that inherent trade offs between individual fairness (calibration) and group fairness are inevitable in real-world data. The paper does not comment on this trade off at all. It will be good if some insights can be included.

4. The paper should also cite and discuss "Fairness through Awareness'' by Dwork et al.

Reviewer #3: This paper is interesting and very complete; just a few points …

I wonder how much the aggregation of diagnoses into ADGs might influence the fairness results. Since ADGs combine conditions with potentially different severity and risk profiles, subgroup differences in ADG coefficients or Shapley values could reflect underlying severity or stage differences rather than true feature-level bias. Do you think using more granular diagnosis codes would change the subgroup fairness patterns you observed?

**********

what does this mean?). If published, this will include your full peer review and any attached files.

**Do you want your identity to be public for this peer review?**  If you choose “no”, your identity will remain anonymous but your review may still be made public.

For information about this choice, including consent withdrawal, please see our Privacy Policy

Reviewer #1: No

Reviewer #2: No

Reviewer #3: No

**********

**Figure resubmission:**

**Reproducibility:**

To enhance the reproducibility of your results, we recommend that authors of applicable studies deposit laboratory protocols in protocols.io, where a protocol can be assigned its own identifier (DOI) such that it can be cited independently in the future. Additionally, PLOS ONE offers an option to publish peer-reviewed clinical study protocols. Read more information on sharing protocols at https://plos.org/protocols?utm_medium=editorial-email&utm_source=authorletters&utm_campaign=protocols

# Author comment: Author response to Decision Letter 1

**DOI:** 10.1371/journal.pdig.0001105.r002

28 Apr 2026

Attachment Submitted filename: Respons letter.docx

# Peer review: Decision Letter 1

**DOI:** 10.1371/journal.pdig.0001105.r003

**Authors:** Krasimira Tsaneva-Atanasova, Mengyu Wang

20 Jul 2026

Response to Reviewers Revised Manuscript with Track Changes Manuscript **Journal Requirements:**

1. For studies involving third-party data, we encourage authors to share any data specific to their analyses that they can legally distribute. PLOS recognizes, however, that authors may be using third-party data they do not have the rights to share. When third-party data cannot be publicly shared, authors must provide all information necessary for interested researchers to apply to gain access to the data. (https://journals.plos.org/digitalhealth/s/data-availability#loc-acceptable-data-access-restrictions)

For any third-party data that the authors cannot legally distribute, they should include the following information in their Data Availability Statement upon submission:

a) A description of the data set and the third-party source

b) If applicable, verification of permission to use the data set

c) Confirmation of whether the authors received any special privileges in accessing the data that other researchers would not have

d) All necessary contact information others would need to apply to gain access to the data

2. Please update your online Competing Interests statement. If you have no competing interests to declare, please state: "The authors have declared that no competing interests exist."

3. If the reviewer comments include a recommendation to cite specific previously published works, please review and evaluate these publications to determine whether they are relevant and should be cited. There is no requirement to cite these works unless the editor has indicated otherwise.

**Additional Editor Comments (if provided):** **Reviewers' Comments:**

**Comments to the Author**

Reviewer #2: (No Response)

**********

publication criteria?

Reviewer #2: Partly

**********

3. Has the statistical analysis been performed appropriately and rigorously?

Reviewer #2: No

**********

4. Have the authors made all data underlying the findings in their manuscript fully available (please refer to the Data Availability Statement at the start of the manuscript PDF file)?

The PLOS Data policy

Reviewer #2: Yes

**********

5. Is the manuscript presented in an intelligible fashion and written in standard English?

Reviewer #2: Yes

**********

Reviewer #2: Some of the major concerns raised by the reviewers still remain in the revised version. In my opinion the most crucial of these are 1) the low predictive accuracy casts doubt on the bias inferences about the data variables. That the predictive accuracy matches the state of the art does not take away the problem 2) possibilities of omitted variables and unobserved confounding make the inferences and interpretations somewhat uncertain 3) most importantly, if there is bias in the underlying data then it is in general impossible to mitigate by negating their effects in predictive machine learning systems. This necessitates making normative decisions about fairness criteria explicit and publicly scrutinised; and paying more attention to the social and medical processes that generate the data in the first place. In that sense the main value of the proposed method is as a diagnostic tool rather than as a predictive tool that mitigates the biases. This needs to be adequately highlighted.

**********

what does this mean?). If published, this will include your full peer review and any attached files.

**Do you want your identity to be public for this peer review?**  If you choose “no”, your identity will remain anonymous but your review may still be made public.

For information about this choice, including consent withdrawal, please see our Privacy Policy

Reviewer #2: No

**********

**Figure resubmission:**

**Reproducibility:**

To enhance the reproducibility of your results, we recommend that authors of applicable studies deposit laboratory protocols in protocols.io, where a protocol can be assigned its own identifier (DOI) such that it can be cited independently in the future. Additionally, PLOS ONE offers an option to publish peer-reviewed clinical study protocols. Read more information on sharing protocols at https://plos.org/protocols?utm_medium=editorial-email&utm_source=authorletters&utm_campaign=protocols

# Author comment: Author response to Decision Letter 2

**DOI:** 10.1371/journal.pdig.0001105.r004

7 Aug 2026

Attachment Submitted filename: Response to Reviewers round 2.docx

# Peer review: Decision Letter 2

**DOI:** 10.1371/journal.pdig.0001105.r005

**Authors:** Krasimira Tsaneva-Atanasova, Mengyu Wang

13 Sep 2026

Refocusing algorithmic fairness on Feature-Level Bias: A Diagnostic approach using Dutch EHR Data

PDIG-D-25-00984R2

Dear MSc. Parsons,

We are pleased to inform you that your manuscript 'Refocusing algorithmic fairness on Feature-Level Bias: A Diagnostic approach using Dutch EHR Data' has been provisionally accepted for publication in PLOS Digital Health.

Before your manuscript can be formally accepted you will need to complete some formatting changes, which you will receive in a follow-up email from a member of our team.

Please note that your manuscript will not be scheduled for publication until you have made the required changes, so a swift response is appreciated.

IMPORTANT: The editorial review process is now complete. PLOS will only permit corrections to spelling, formatting or significant scientific errors from this point onwards. Requests for major changes, or any which affect the scientific understanding of your work, will cause delays to the publication date of your manuscript.

If your institution or institutions have a press office, please notify them about your upcoming paper to help maximize its impact. If they'll be preparing press materials, please inform our press team as soon as possible -- no later than 48 hours after receiving the formal acceptance. Your manuscript will remain under strict press embargo until 2 pm Eastern Time on the date of publication. For more information, please contact digitalhealth@plos.org.

Thank you again for supporting Open Access publishing; we are looking forward to publishing your work in PLOS Digital Health.

Best regards,

Krasimira Tsaneva-Atanasova

Academic Editor

PLOS Digital Health

***********************************************************

**Additional Editor Comments (if provided):**

**Reviewer Comments (if any, and for reference):**

Reviewer's Responses to Questions

**Comments to the Author**

Reviewer #2: All comments have been addressed

**********

publication criteria?

Reviewer #2: Yes

**********

3. Has the statistical analysis been performed appropriately and rigorously?

Reviewer #2: Yes

**********

4. Have the authors made all data underlying the findings in their manuscript fully available (please refer to the Data Availability Statement at the start of the manuscript PDF file)?

The PLOS Data policy

Reviewer #2: Yes

**********

5. Is the manuscript presented in an intelligible fashion and written in standard English?

Reviewer #2: Yes

**********

Reviewer #2: I am satisfied with the revision

**********

what does this mean?). If published, this will include your full peer review and any attached files.

**Do you want your identity to be public for this peer review?**  If you choose “no”, your identity will remain anonymous but your review may still be made public.

For information about this choice, including consent withdrawal, please see our Privacy Policy

Reviewer #2: No

**********
